# The steroid hormone 20-hydroxyecdysone induces lipophagy *via* the brain-adipose tissue axis by promoting the adipokinetic hormone pathway

**DOI:** 10.1016/j.jbc.2025.108179

**Published:** 2025-01-10

**Authors:** Yan-Xue Li, Yan-Li Li, Xiao-Pei Wang, Tian-Wen Liu, Du-Juan Dong, Jin-Xing Wang, Xiao-Fan Zhao

**Affiliations:** Shandong Provincial Key Laboratory of Animal Cells and Developmental Biology, School of Life Sciences, Shandong University, Qingdao, China

**Keywords:** 20-hydroxyecdysone, adipokinetic hormone, adipokinetic hormone receptor, glucose, lipophagy

## Abstract

Lipophagy is a way to degrade lipids; however, the molecular mechanisms are not fully understood. Using the holometabolous lepidopteran insect *Helicoverpa armigera*, cotton bollworm, as a model, we revealed that the larval fat body undergoes lipophagy during metamorphosis, and lipophagy is essential for metamorphosis. The steroid hormone 20-hydroxyecdysone (20E) induced lipophagy by promoting the expression of the peptide hormone adipokinetic hormone (AKH, the insect analog of glucagon) and the adipokinetic hormone receptor (AKHR). *Akh* was highly expressed in the brain and *Akhr* was expressed in various tissues. The 20E upregulated the expression of *Akh* and *Akhr* by its nuclear receptor EcR during metamorphosis. AKH and AKHR increased glucose levels *via* gluconeogenesis and promoted lipophagy. The high glucose level induced acetylation of FOXO and nuclear localization to promote the expression of lipases and autophagy genes. Thus, the steroid hormone 20E induced lipophagy *via* the brain–adipose tissue axis by promoting the AKH pathway, which presented nutrients and energy to pupal and adult development during insect metamorphosis after feeding stops.

Glycolipid metabolism is essential to animal and human growth and health. Glycolipid metabolism is regulated by various hormones. One of the hormones is glucagon, a vital polypeptide hormone synthesized by the α cells of the pancreas in mammals. Glucagon binds its receptor, a G protein-coupled receptor (glucagon receptor, GCGR), to regulate fat and glucose mobilization by an antagonistic effect against insulin (1, 2, 3). Glucagon raises glucose in plasma by breaking glycogen into free sugars (4) and gluconeogenesis (5). Glucagon also induces lipolysis in the liver (6). In the absence of glucagon signaling, glucose metabolic disorder (1), hepatic lipolysis, and autophagy are halted, resulting in the accumulation of hepatic fat. A research report shows that the lipolytic effect of glucagon may be partly mediated by lipophagy (7).

The typical pathway of lipolysis is catalyzed by three main lipases: adipocyte triglyceride lipase (ATGL), hormone sensitive lipase, and monoacylglycerol lipase (8, 9, 10, 11). Besides this lipolysis process, lipophagy can also break down lipids, a selective autophagy targeting lipid droplets (LDs). Autophagy selectively isolates intracellular contents by double-membrane structures of autophagosomes through a highly coordinated network of proteins; then, the fusion of the autophagosome with the lysosome leads to the degradation of the autophagy cargo (12). Lipophagy begins with the recognition of cargo by the autophagosomal membrane through interaction with microtubule-associated protein 1 light chain 3 (LC3, autophagy-related gene 8, ATG8/LC3), followed by autophagosomes engulfing LDs and fusing with a lysosome to form an autolysosome; then, lipids in LDs are hydrolyzed by lysosomal lipases (13). Sequestosome 1 (SQSTM1/p62) is a selective autophagy receptor involved in lipophagy by bridging LDs or LD-containing aggresomes with the phagophores. p62 and LDs codegraded in the lysosomes, and p62 accumulates when autophagy is blocked (14). In rat hepatocytes, lipolysis targets larger-sized LDs to produce some small LDs, and the small LDs are degraded by lipophagy, confirming the synergistic relationship between lipolysis and lipophagy (15). There are many more links between lipolysis and lipophagy, such as ATGL, which induces lipophagy by interacting with LC3 (16) and promotes lipophagy to regulate liver LD catabolism through SIRT1 (17). ATGL is necessary for lipolysis and plays a vital role in lipophagy. Lipophagy is essential to lipid metabolism; the deletion of oxysterol-binding protein (OSBP)-related protein 8 (ORP8), a lipophagy receptor, causes liver lipid clearance defects in mice (18). The steroid hormone glucocorticoids (GCs) are vital in glycolipid metabolism. GCs are synthesized and released from the adrenal cortex to act on multiple tissues, including adipose tissue, the liver, and the skeletal muscles (19). GCs have similar functions as glucagon in glycolipid metabolism, such as elevating plasma glucose by glycogenolysis and gluconeogenesis (20) and induce lipolysis in adipose tissue (21). The glucocorticoid receptor plays a crucial role in lipid metabolism, and ginsenoside compound K (CK, a natural small molecule with a steroid-like structure) binds to glucocorticoid receptor to induce lipophagy (22). Glucagon levels can be increased by GCs (20). The synthetic compound dexamethasone with GC activity results in a higher proportion of glucagon (23, 24). Nevertheless, the relationship among steroid hormones, the peptide hormone glucagon, and lipophagy is not fully understood.

In insects, the adipokinetic hormone (AKH) corresponds to mammalian glucagon (25, 26). AKH is biosynthesized and secreted from the corpora cardiaca (CC) (27). The primary function of AKH is the mobilization of stored macromolecules from the fat body to raise levels of circulating metabolites (trehalose, diacylglycerols, proline, and so on) to increase circulating levels of trehalose and lipids (28, 29, 30). AKH promotes lipid hydrolysis of the fat body through a G protein-coupled receptor (AKH receptor, AKHR) (31) and lipase (32). The fat body of insects can be regarded as the functional equivalent of mammalian liver and white adipose tissue (33). Under energy-demanding conditions, after feed stops during metamorphosis, the fat body is hydrolyzed to supply lipids for adult development (34). The steroid hormone 20-hydroxyecdysone (20E) promotes macroautophagy and apoptosis of the fat body (35, 36, 37). Both 20E and AKH promote lipolysis; however, the relationship between 20E and AKH and the mechanism by which they regulate lipophagy of the fat body during metamorphosis is unclear.

In the present study, we used the lepidopteran insect *Helicoverpa armigera*, cotton bollworm, as a model to reveal the relationship between 20E and AKH and the mechanism by which they regulate lipophagy. We determined 20E upregulated *Akh* and *Akhr* expression, respectively, to promote gluconeogenesis to elevate glucose levels, and the high glucose level induced forkhead box O (FOXO) acetylation for gene expression to induce lipophagy of the fat body during insect metamorphosis.

## Results

### Lipophagy occurs in the fat body during metamorphosis

**Figure 1:**
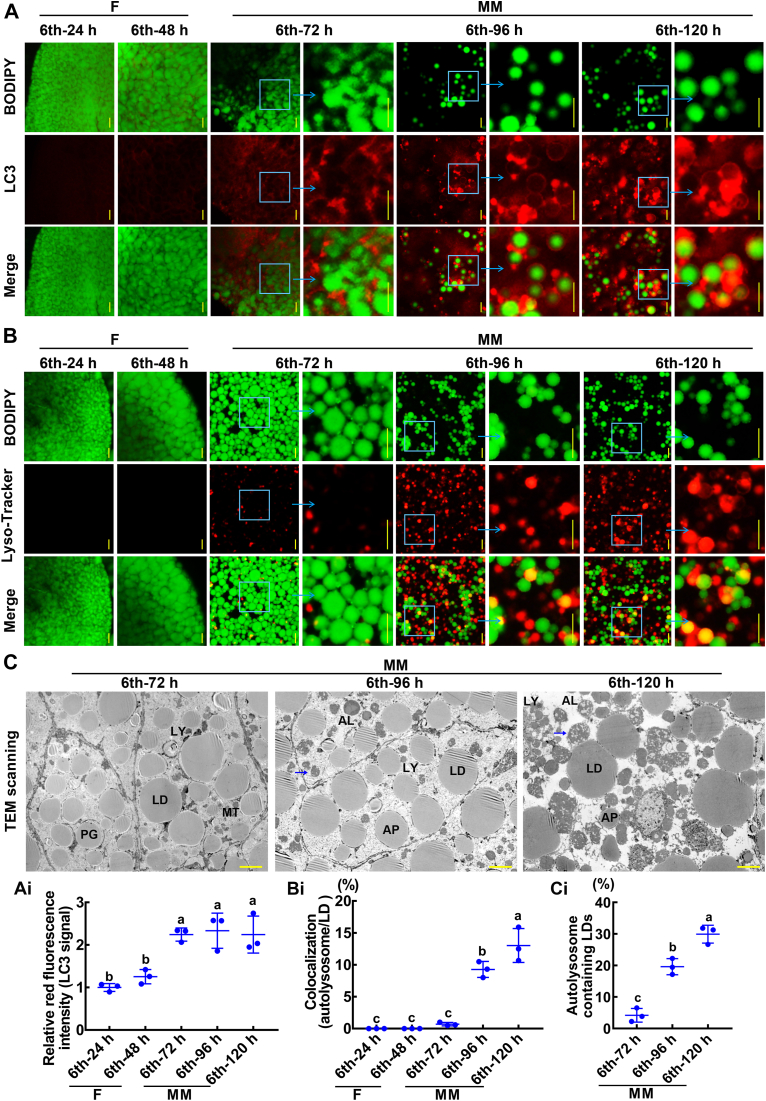
**The lipophagy developmental profiles of the fat body.***A*, colocalization of LDs and LC3. BODIPY (5 μM for 30 min, *green* fluorescence) indicates LDs. LC3 (*red* fluorescence) indicates autophagosomes. Merge was the overlap of *red* and *green* fluorescence. F: feeding; MM: metamorphic molting. The bars represent 50 μm. *Ai*, statistical analysis of the LC3 signal intensity by ImageJ software. *B*, colocalization of LDs and lysosomes. BODIPY (5 μM for 30 min, *green fluorescence*). Lyso-Tracker (50 nM for 10 min, *red fluorescence*). Merge was the overlap of *red* and *green* fluorescence. The scale bars represent 50 μm. *Bi*, the ratio of lipophagy LDs (*yellow*) to total LDs (*green*). *C*, TEM images of fat body during metamorphosis. *Blue arrows* indicate that LD is entrapped in the lysosome. The bars represent 50 μm. LD: lipid droplet; PG: phagophore; AP: autophagosome; LY: lysosome; AL: autolysosome. *Ci*, the ratio of autolysosome containing LDs to total LDs. The statistical analysis was performed using three independent replicates by ANOVA. LC3, microtubule-associated protein 1 light chain 3; TEM, transmission electron microscopy.

To determine the lipophagy occurring in the fat body during metamorphosis, we performed colocalization of LDs, LC3, and lysosomes. The colocalization of LDs (green) and LC3 (red) was undetectable during the feeding stage (sixth instar 24 h and sixth instar 48 h) but was observed during the metamorphotic stage (sixth instar 72 h to 120 h) (Fig. 1, *A* and *Ai*). Moreover, the colocalization of LDs (green) and lysosomes (red) was observed during the metamorphotic stage (Fig. 1, *B* and *Bi*). We observed that the LDs were enclosed by double-membrane vesicles, a feature of autophagosomes, and LDs in the lysosomes by transmission electron microscopy (TEM) (Fig. 1, *C* and *Ci*). These data indicated that lipophagy in the fat body occurs during metamorphosis.

### Lipophagy is essential for metamorphosis

**Figure 2:**
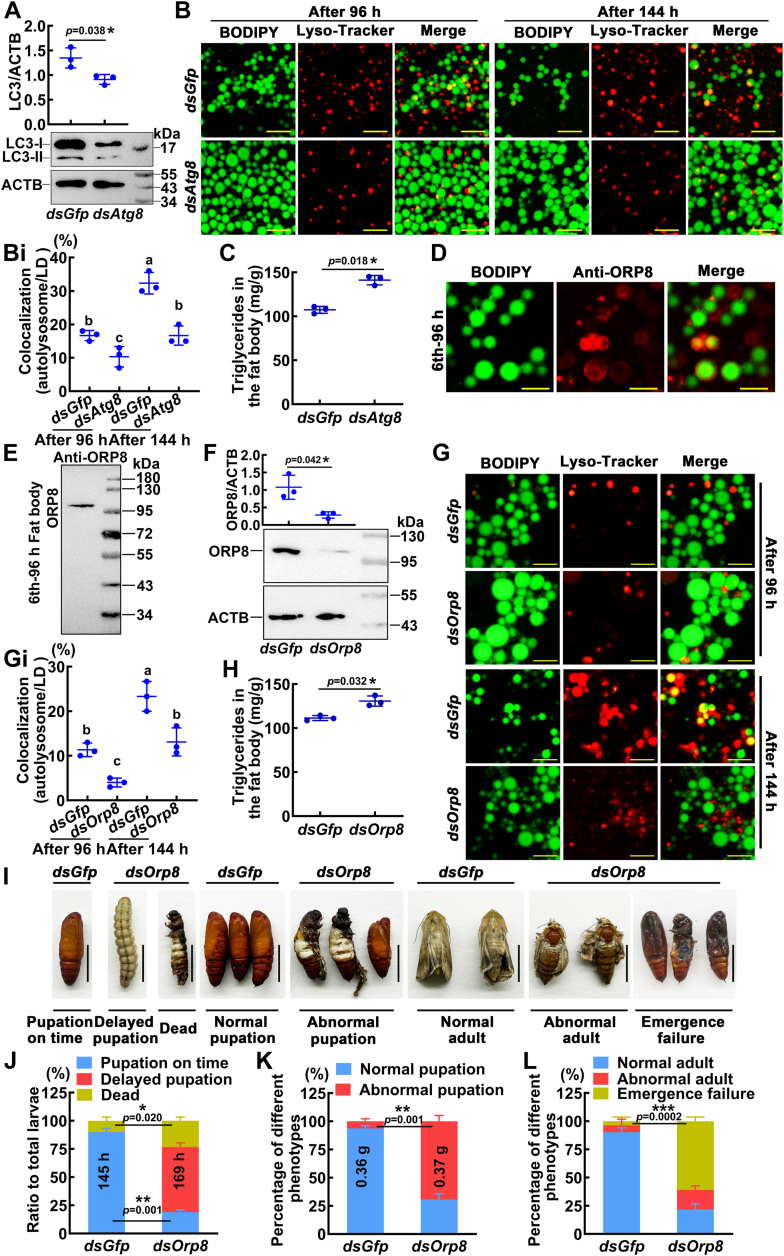
**Knockdown of *Atg8* and *Orp8* blocked lipophagy.***A*, interference efficiency was detected by Western blotting. *B*, the colocalization of lysosomes and LDs in fat body cells after *dsAtg8* injection. Lysosomes were stained with Lyso-Tracker (*red*), and LDs were stained with BODIPY (*green*). *Yellow* indicates examples of Lyso-Tracker-positive structures containing LDs. The ruler represents 50 μm. *Bi*, statistical analysis of autolysosome numbers in (*B*). *C*, the levels of triglycerides in the fat body after *Atg8* knockdown. *D*, the location of ORP8 in fat body cells. *Red fluorescence* represents ORP8; *green fluorescence* represents LDs. The scale bar is 50 μm. *E*, the specificity of the ORP8 antibody in the fat body was analyzed by Western blotting. *F*, the expression of ORP8 was analyzed by Western blotting. *G* and *Gi*, the colocalization of LDs and lysosomes was detected after *Orp8* knockdown. The ruler represents 50 μm. *H*, the levels of triglycerides in the fat body after *Orp8* knockdown. *I*, phenotypes after *dsOrp8* injection. Bars represent 1 cm. *dsGfp* was used as a control. *J*, statistical analysis of the phenotypes in (*I*). The pupation time was sixth instar 0 h larvae to pupae. *K*, the average weight of pupae and pupal phenotypes. *L*, percentage of different phenotypes from pupae to adults. The bars indicate the mean ± SD. *p* values and *asterisks* indicate differences by two-tailed Student's *t* test (∗*p* < 0.05, ∗∗*p* < 0.01). The different lowercase letters indicate significant differences in ANOVA (*p* < 0.05). LD, lipid droplet; ORP8, oxysterol-binding protein-related protein 8.

To examine the function of lipophagy in insect metamorphic development, we knocked down autophagy-related genes *via* injection of dsRNA into sixth instar 6 h larvae. When p62 was knocked down, the level of p62 in the fat body was significantly decreased (Fig. S1*A*), and the fusion of lysosomes and LDs was blocked (Fig. S1, *B* and *Bi*), resulting in the accumulation of triglycerides (TGs) in the fat body (Fig. S1*C*). Knockdown of p62 resulted in death in 72% of the individuals (Fig. S1, *D* and *E*), suggesting that p62 regulated lipophagy and promoted metamorphosis. After the knockdown of the autophagy-related gene *Atg8*, LC3 levels in the fat body were significantly reduced (Fig. 2*A*), and lysosome fusion with LDs was hindered (Fig. 2, *B* and *Bi*). The decrease in LC3 levels caused the accumulation of TG compared to the control group (Fig. 2*C*). The ORP8 presented punctate or ring-like signals and colocalized to LDs (Fig. 2*D*). We performed a Western blot ORP8, which was detected as a single band using the anti-ORP8 antibody in the fat body of sixth-96 h (Fig. 2*E*). When the expression level of ORP8 was significantly reduced (Fig. 2*F*), the colocalization of lysosome and LDs was hindered (Fig. 2, *G* and *Gi*), resulting in the increase of TG levels in the fat body (Fig. 2*H*). After *Orp8* expression was significantly reduced by RNAi (Fig. 2*I*), 58% of the larvae showed a delayed pupation phenotype, the larval pupation time was delayed by 24 h (Fig. 2*J*), there was no difference in average pupa weight, and 69% of the pupae showed abnormal phenotypes (Fig. 2*K*), and 61% of the pupae failed to emerge during development (Fig. 2*L*). The data indicated that lipophagy is essential for metamorphosis.

### 20E promoted the transcription of *Akh* and *Akhr via* EcR

**Figure 3:**
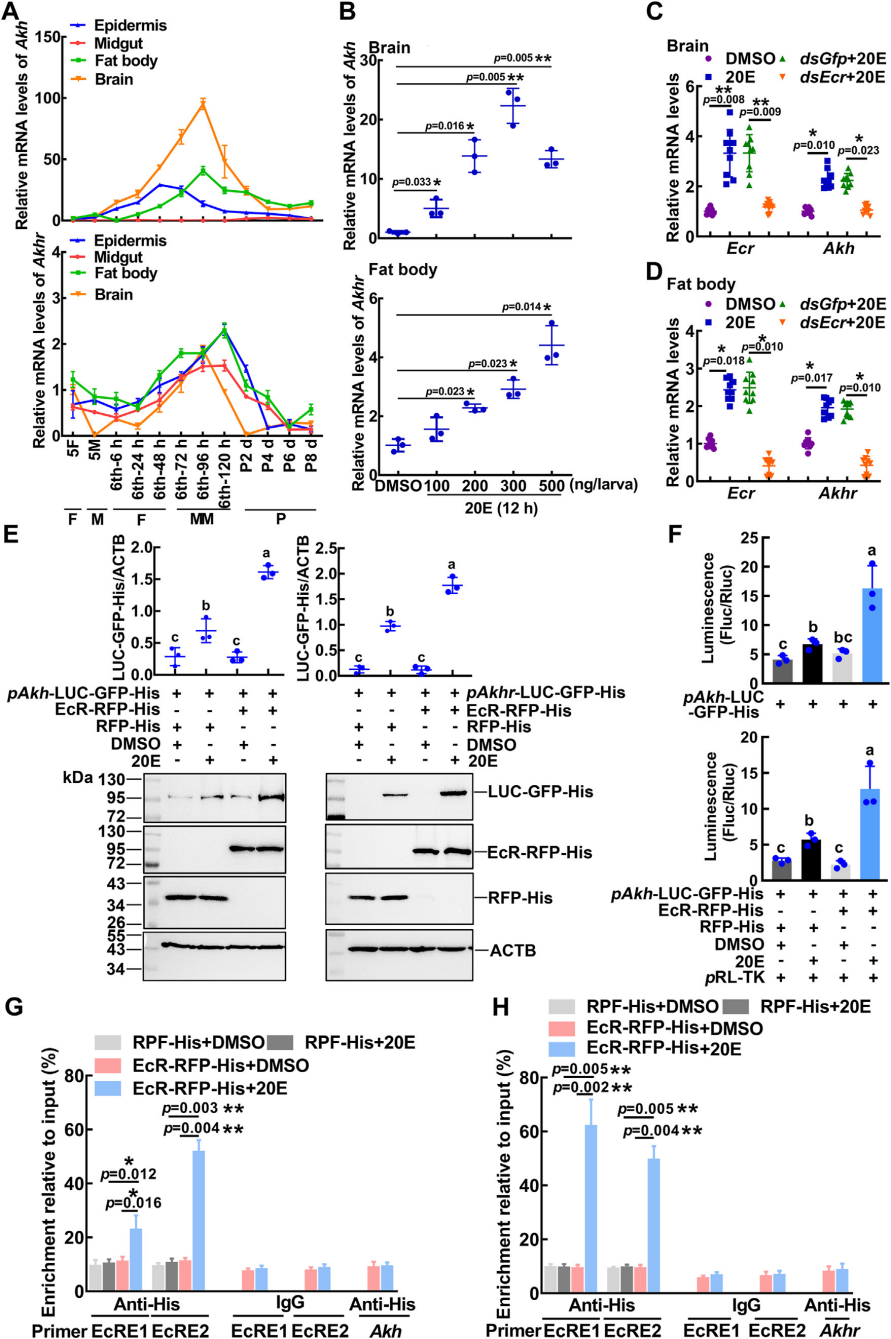
**20E promoted the expression of *Akh* and *Akhr via* EcR.***A*, the expression profiles of *Akh* and *Akhr* in the epidermis, midgut, fat body, and brain were detected using qRT-PCR. 5F: fifth instar feeding larvae; 5M: fifth instar molting larvae; sixth-6 h to 120 h: sixth instar larvae at different stages; P2 d to P8 d: 2 to 8-day-old pupae. F: feeding; M: molting; MM: metamorphic molting; P: pupae. *B*, the expression of *Akh* and *Akhr* under stimulation with different concentrations of 20E for 12 h. *C* and *D*, knockdown of *Ecr* by *dsEcr* (2 μg/larva) followed by stimulation with 20E (500 ng/larva) for 12 h to detect the expression of *Akh* and *Akhr*. *E*, Western blotting showed the expression of the *pAkh*/*pAkhr*-LUC-GFP-His reporter plasmid (LUC-GFP-His) under overexpression of EcR or RFP with 20E or DMSO treatment. *F*, the fluorescence of the firefly (Fluc) represents reporting activity, and the fluorescence of renilla (Rluc) was used as an internal reference to eliminate background. *G*, ChIP assay showed that 20E promotes *Akh* expression *via* EcR binding to EcRE1 and EcRE2. The primers for EcRE are the sequences containing EcRE in the *Akh* promoter region, respectively. Primer *Akh* targeting the *Akh* coding DNA sequence (CDS) was used as a control. *H*, ChIP assays confirmed that 20E increases the binding of EcR to EcRE1 and EcRE2. The primers EcRE are the sequences containing EcRE in the *Akhr* promoter region. Primer *Akhr* targeting the *Akhr* coding DNA sequence (CDS) was used as a control. Data are the mean ± SD of three replicates. ∗*p* < 0.05, ∗∗*p* < 0.01 (two-tailed Student's *t* test). The comparison among multiple sets of data was performed by analysis of variance (ANOVA). The different lowercase letters show significant differences. 20E, 20-hydroxyecdysone; ChIP, chromatin immunoprecipitation; qRT-PCR, quantitative real-time reverse transcription PCR; DMSO, dimethyl sulfoxide.

To explore the genes involved in lipophagy, the developmental expression profiles of *Akh* and *Akhr* were analyzed. *Akh* was highly expressed in the brain at the metamorphic stage, and *Akhr* was expressed in various tissues and highly expressed at the metamorphic stage (Fig. 3*A*).

To investigate the regulatory effect of 20E on *Akh* and *Akhr*, we injected 20E into the larval hemocoel. The expression of *Akh* and *Akhr* was increased in a time- and 20E concentration-dependent manner (Fig. 3*B*). The binding sites of EcR (EcRE) were found in the promoter regions of *Akh* and *Akhr* by predictive analysis in the JASPAR transcription factor database (Fig. S2*A*). The expression of *Akh* and *Akhr* decreased after knockdown of *Ecr* (Fig. 3, *C* and *D*), suggesting that 20E promoted *Akh* and *Akhr* expression *via* EcR.

To determine whether 20E directly promotes the transcription of *Akh* and *Akhr* by EcR, we constructed luciferase and green fluorescent protein reporter plasmids of the *Akh* and *Akhr* promoters (containing the 5′-untranslated region, *pAkh*/*pAkhr*-LUC-GFP-His) (Fig. S2, *B* and *C*). Western blotting showed that the expression of the reporter gene (LUC-GFP-His) of *pAkh*-LUC-GFP-His and *pAkhr*-LUC-GFP-His was significantly upregulated under 20E induction when EcR-RFP-His was overexpressed (Fig. 3*E*). Similarly, when EcR-RFP-His was overexpressed, the luciferase activity of *pAkh*-LUC-GFP-His and *pAkhr*-LUC-GFP-His showed a strong increase in a 20E-dependent manner (Fig. 3*F*). The chromatin immunoprecipitation (ChIP) assay showed that the sequence motifs of EcRE1 and EcRE2 were the binding element sequences in the *Akh* promoter (Fig. 3*G*). The ChIP assay showed that EcR-RFP-His enriched more binding element sequences (EcRE1 and EcRE2) in the *Akhr* promoter under 20E treatment than in the dimethyl sulfoxide (DMSO) treatment control (Fig. 3*H*). These results suggested that 20E promotes the transcription of *Akh* and *Akhr* through EcR.

By *in situ* hybridization analysis, we detected strong signals of mRNA of AKH in the CC and brain (Fig. S3), suggesting AKH is expressed not only in the CC but also in the brain.

### AKH and AKHR promoted fat body cell lipophagy and apoptosis

**Figure 4:**
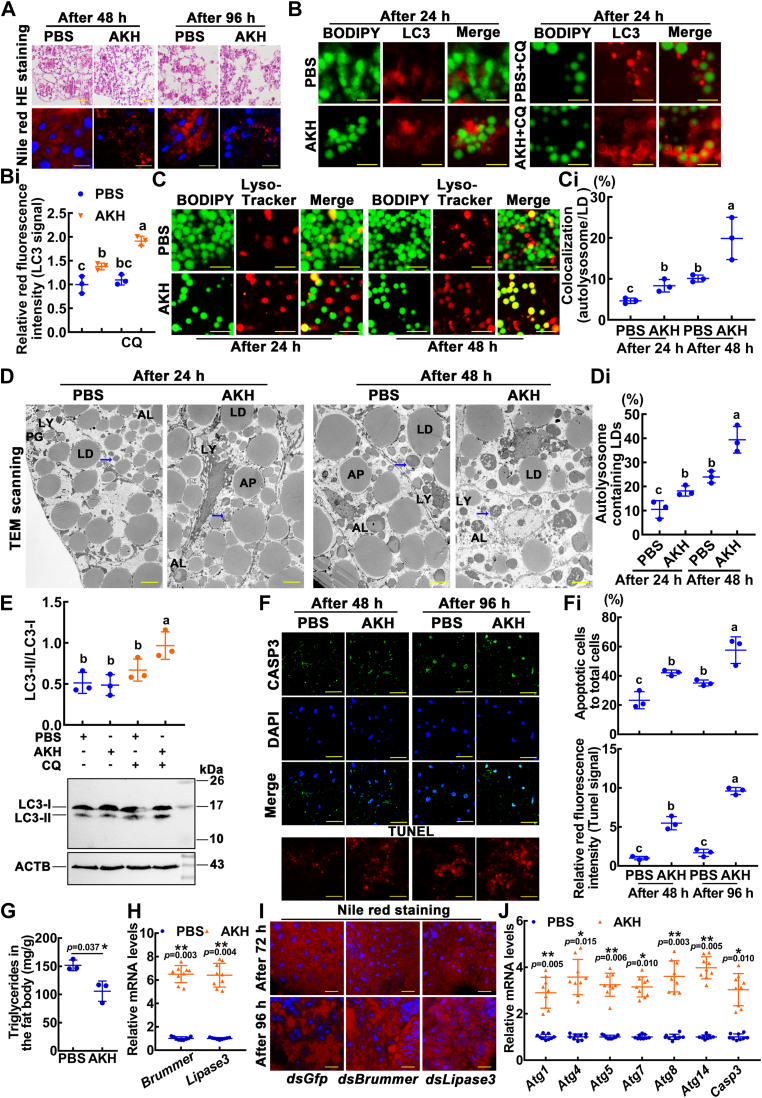
**AKH injection promoted fat body cell lipophagy and apoptosis.***A*, H&E staining showed fat body morphology. Nile red staining showed LDs. Samples were observed after the first injection (at sixth-72 h) of AKH (24 ng/larva = 100 nM) for 48 h and 96 h. PBS was used as a control. The rulers represent 20 μm in the H&E staining and 50 μm in Nile red. *B*, the colocalization of LC3 and LDs in fat body cells. BODIPY (5 μM for 30 min, *green fluorescence*) indicates LDs. LC3 indicates autophagosomes. CQ: chloroquine, 10 μM for 24 h. The ruler represents 50 μm. *Bi*, the relative red fluorescence intensity showed LC3 protein by immunofluorescence staining. *C*, the colocalization of lysosomes and LDs in fat body cells. Costaining with 50 nM Lyso-Tracker and 5 μM BODIPY (*green*) indicate the induction of lipophagy (*yellow*). The ruler represents 50 μm. *Ci*, statistical analysis of fat body cell lipophagy. *D*, TEM observation after injection with AKH in the fat body. The *blue arrow* shows that LD is entrapped in the lysosome. The bars represent 50 μm. LD: lipid droplet; PG: phagophore; AP: autophagosome; LY: lysosome; AL: autolysosome. *Di*, The ratio of autolysosome containing LDs to total LDs. *E*, Western blotting and statistical analysis showing LC3-II protein levels. CQ: chloroquine, 10 μM for 24 h. The samples were obtained 48 h after the first injection. *F*, CASP3 location in the fat body after AKH injection. Antibodies against CASP3 were used as the primary antibody, and antibodies against rabbit IgG Alexa Fluor 488 were used as the secondary antibody. *Green* fluorescence indicates CASP3, and *blue* fluorescence indicates nuclei are stained with DAPI. TUNEL staining shows apoptosis. The rulers represent 50 μm. *Fi*, the ratio of apoptotic cells (*green*) to total cells (*blue*). The *red* fluorescence intensity (tunnel signal) was counted by ImageJ. *G*, measurement of triglyceride levels in the fat body. The samples were obtained 48 h after the first injection. *H*, qRT-PCR showing the mRNA levels of lipase genes. *I*, Nile *red* staining showed fat body LDs after knockdown of *Brummer* or *Lipase3*. Images were observed after the first injection of dsRNA (sixth-6 h) for 72 h and 96 h. The scale bar represents 50 μm. *J*, qRT-PCR analysis of the mRNA levels of autophagy and apoptosis (PCD-related) genes. Statistical analysis was conducted using ANOVA or Student's *t* test. The different lowercase letters show significant differences. *Asterisks* denote significant differences (∗*p* < 0.05, ∗∗*p* < 0.01). AKH, adipokinetic hormone; LD, lipid droplet; LC3, microtubule-associated protein 1 light chain 3; PCD, programmed cell death; TEM, transmission electron microscopy; qRT-PCR, quantitative real-time reverse transcription PCR; DAPI, 4′,6-diamidino-2-phenylindole.

To explore the role of AKH in lipophagy, AKH (24 ng/larva) was injected into the sixth instar 72 h larvae hemocoel. AKH caused severe dissociation of the fat body, as shown by H&E staining. Nile red staining showed that the LDs were broken down into small droplets (Fig. 4*A*). Moreover, AKH promoted LC3-positive membrane engulfment of LDs (Fig. 4, *B* and *Bi*). AKH induced an increase in the colocalization of lysosomes (red) and LDs (green), which indicated the induction of lipophagy (yellow) by AKH (Fig. 4, *C* and *Ci*). The TEM showed that the number of typical autophagosomes and autolysosomes, which had two or single-membraned and contained LD, increased in the fat body after AKH injection compared with the PBS control (Fig. 4, *D* and *Di*). Western blotting further revealed that the transformation of LC3-I to LC3-II (phosphatidyl ethanolamine form) was increased by AKH injection (Fig. 4*E*). Compared with the PBS, the injection of AKH resulted in a significant reduction in p62 levels (Fig. S4*A*). Interference with *Atg1* obstructed AKH-induced degradation of LC3-II and p62, and colocalization of lysosomes and LDs (Fig. S4, *B*–*E*). Immunohistochemistry showed that CASP3 was mainly located in the cytoplasm of the larval fat body after injecting PBS; however, CASP3 was strongly localized in the nuclei in the AKH-injected fat body. TUNEL staining revealed the increase of apoptosis in the fat body cells after AKH injection (Fig. 4, *F* and *Fi*). AKH treatment resulted in a decrease in TG levels (Fig. 4*G*). Brummer and acid lipase-1 promoted lipolysis in *the Bombyx mori* fat body (38). AKH treatment resulted in the upregulated expression of *Brummer* and *Lipase3* (homolog of *Brummer* and *acid lipase-1* of *B. mori*) (Fig. 4*H*). Knockdown of *Brummer* and *Lipase3* blocked the dissociation of the fat body (Figs. 4*I*, S4, *F* and *G*). The mRNA levels of *Atgs* and *Casp3* (programmed cell death [PCD]-related genes) increased after AKH induction (Fig. 4*J*). The data indicated that AKH promotes fat body cell lipophagy and apoptosis.

**Figure 5:**
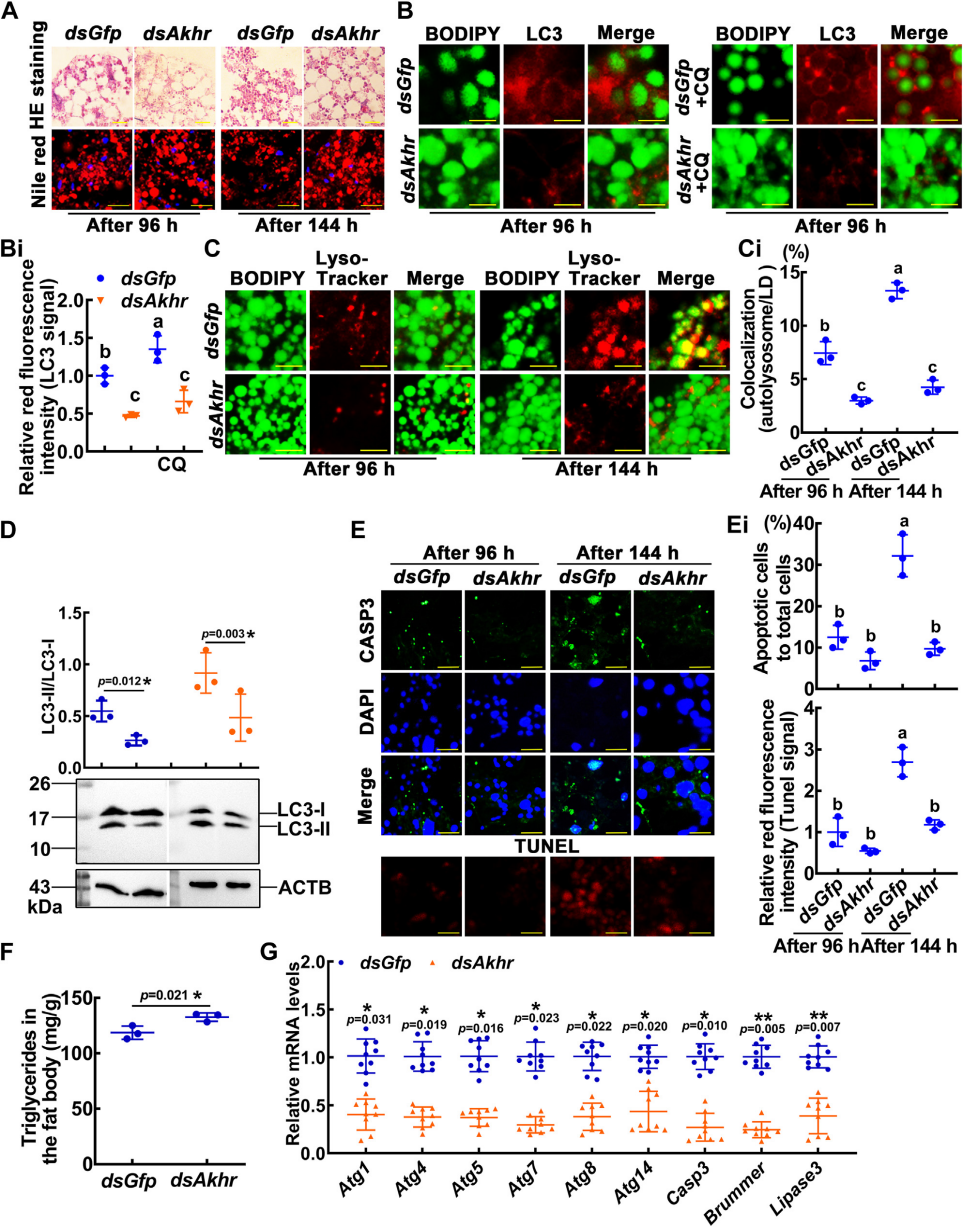
**AKHR promoted lipophagy in fat body cells.***A*, H&E- and Nile red-stained fat body after the knockdown of *Akhr*. *dsGfp* was used as a control. Samples were taken 96 h or 144 h after the first injection (at sixth-6 h) of dsRNA (500 ng/larva, a total of four injections, 24 h apart). *Red fluorescence* indicates LDs, and *blue* fluorescence indicates nuclei. The rulers represent 20 μm in the H&E staining and 50 μm in the Nile red staining. *B*, the colocalization of LC3 (*red*) and LDs (*green*). The ruler represents 50 μm. *Bi*, quantification of the *red* fluorescence intensity of the LC3 protein. *C*, the colocalization of lysosomes (*red* fluorescence, 50 nM LysoTracker for 10 min) and LDs (*green*, 5 μM BODIPY for 30 min). The *yellow* color represents the colocalization of autolysosomes and LDs. The bars represent 50 μm. *Ci*, the ratio of autolysosomes in lipophagy. *D*, LC3-II levels were visualized by Western blotting with antibodies against LC3. CQ: chloroquine, 10 μM for 24 h. Samples were taken 96 h after the first injection of dsRNA. *E*, the CASP3 and TUNEL signals in the fat body after *Akhr* knockdown. *Green* fluorescence indicates CASP3 is detected by an antibody against CASP3. *Blue* fluorescence indicates nuclei are stained with DAPI. *Red* fluorescence indicates the TUNEL signal. Scale bar represents 25 μm. *Ei*, the ratio of apoptotic cells (*green*) to total cells (*blue*). The *red* fluorescence intensity (tunnel signal) was counted by ImageJ. *F*, measurement of triglyceride levels after *Akhr* knockdown in the fat body. *G*, qRT-PCR showed the mRNA levels of lipase and PCD-related genes. Error bars show the mean ± SD. The significant difference was calculated by Student's *t* test (∗*p* < 0.05; ∗∗*p* < 0.01) or one-way analysis of variance (ANOVA, *p* < 0.05). AKHR, adipokinetic hormone receptor; LD, lipid droplet; LC3, microtubule-associated protein 1 light chain 3; PCD, programmed cell death; qRT-PCR, quantitative real-time reverse transcription PCR; DAPI, 4′,6-diamidino-2-phenylindole.

For determination of the AKH promoted lipophagy, we knocked down *Akhr* by injecting *dsAkhr* into sixth instar 6 h larvae hemocoel. The expression level of *Akhr* was significantly reduced (Fig. S4*H*), the dissociation of the fat body was blocked, and the LDs were kept large and dense (Fig. 5*A*). Knockdown of *Akhr* prevented the LC3-positive membranes from engulfing LDs (Fig. 5, *B* and *Bi*) and the colocalization of LDs and lysosomes (Fig. 5, *C* and *Ci*). Western blotting showed that the formation of LC3-II was reduced and the degradation of p62 was hindered after *Akhr* knockdown (Figs. 5*D* and S5*A*). The CASP3 and TUNEL signals were very light compared to the control's (Fig. 5, *E* and *Ei*). *Akhr* knockdown resulted in TG accumulation (Fig. 5*F*), and the expression levels of lipase genes and PCD-related genes decreased (Fig. 5*G*). AKH promoted lipophagy; however, after knockdown of *Akhr*, AKH did not promote the colocalization of LDs and lysosomes, but caused TG accumulation, compared with larvae injected with *dsGfp* plus AKH (Fig. S5, *B*–*D*). These results suggested that AKHR participates in lipophagy.

### AKH and AKHR elevated hemolymph glucose levels to support metamorphosis

The increase of the hemolymph glucose was induced by 20E (Fig. S5*E*). The mRNA levels of the key enzymes for gluconeogenesis, glucose-6-phosphatase (*G6pase*) and phosphoenolpyruvate carboxykinase (*Pepck*), were highly increased after AKH treatment (Fig. S6*A*). To determine the role of the AKH pathway in elevated glucose levels, we assessed the hemolymph glucose level after AKH and *dsAkhr* injection. Glucose levels increased after AKH injection (Fig. S6*B*). Pupation time showed no significant change after AKH injection (Fig. S6*C*). AKH significantly decreased pupa weight for an average of 0.03 to 0.05 g (Fig. S6*D*). AKH injection resulted in small pupae and metamorphic failure, in which 47.8 to 56.5% of larvae grew to small pupae, 26.7 to 44.4% of larvae died, and more than 80% of pupae failed to eclose normally (Fig. S6, *E*–*G*). Consistently, when *Akhr* expression was significantly reduced by RNAi (Fig. S6*H*), the expression of *G6pase* and *Pepck* was decreased (Fig. S6*I*), and the glucose level was also significantly decreased compared with that in the larvae injected with *dsGfp* (Fig. S6*J*). The larval pupation time was delayed by 19 h (Fig. S6*K*), and pupa weight had no significant effect after *dsAkhr* injection (Fig. S6*L*). After *Akhr* knockdown, 57% of larvae showed a delayed pupation phenotype, 73% of the pupae showed abnormal phenotypes, and 84% of the pupae failed to emerge during development (Fig. S6, *M*–*P*). After *Akhr* was significantly knocked down in the fat body, AKH could not upregulate the expression of the *G6pase* and *Pepck* (Fig. S7*A*), and the glucose level was also significantly decreased compared with that in the larvae injected with *dsGfp* plus AKH (Fig. S7*B*). These results suggested that AKH and AKHR increase hemolymph glucose levels by inducing the expression of gluconeogenetic genes, which is vital for metamorphosis.

### High glucose levels promoted lipophagy

**Figure 6:**
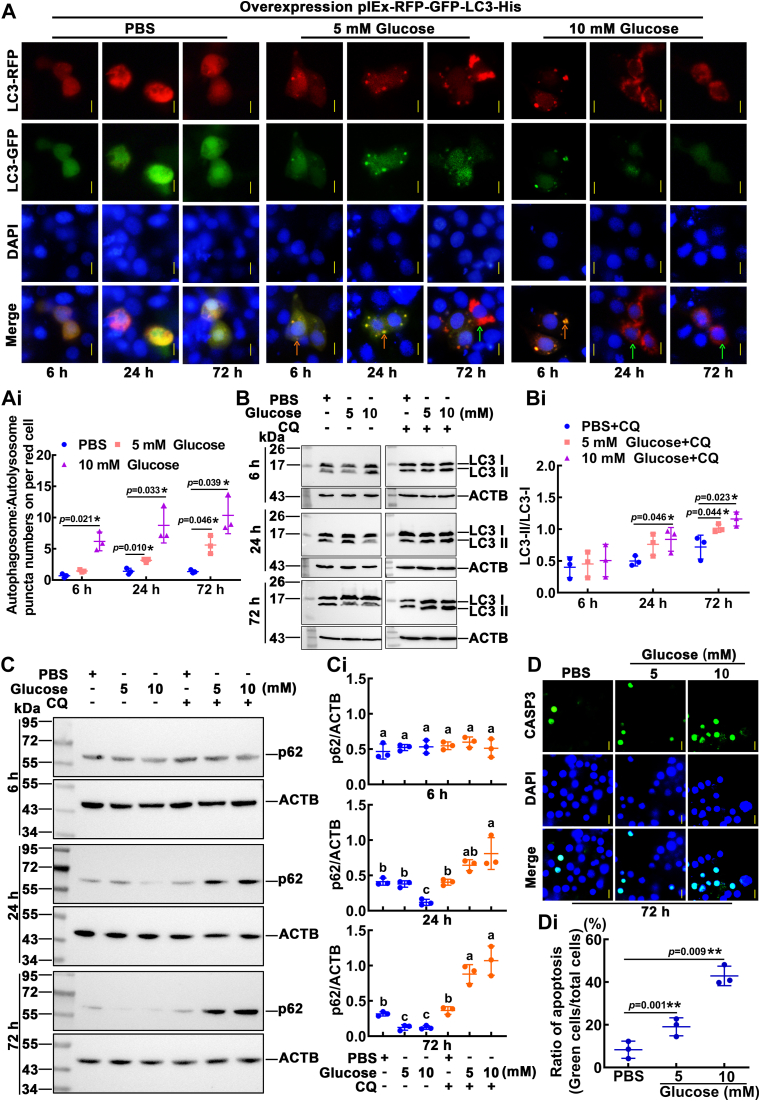
**Glucose promoted autophagy and apoptosis, as detected by autophagic flux and CASP3 activity.***A*, after glucose treatment, autophagic flux was detected in HaEpi cells, and PBS was used as the control. *Green* and *red* fluorescence indicate the autophagic flux from autophagosomes (*red* and *green* fluorescence) to autolysosomes (*red* fluorescence only). DAPI: nuclei stained as *blue* fluorescence. The *yellow bars* represent 20 μm. The *orange arrows* represent autophagosome puncta. The *green arrows* represent autolysosome puncta. *Ai*, the number of autophagosome puncta and autolysosome puncta in cells successfully transfected with pIEx-GFP-RFP-LC3 was counted. *B*, Western blotting showed the changes in LC3-II after treatment with different concentrations and times of glucose. CQ: chloroquine, 10 μM for 24 h. *Bi*, quantification of the data in (*B*). *C*, Western blotting showed the changes in p62 after treatment with different concentrations and times of glucose. CQ: chloroquine, 10 μM for 24 h. *Ci*, quantification of the data in (*C*). *D*, detection of cell apoptosis by CASP3 activity. *Green* fluorescence represents CASP3 activity, assessed using a CASP3 activity detection kit. *Blue* fluorescence indicates DAPI-stained nuclei. Merge: the superimposed images of the *green* and *blue* fluorescence. The scale bar represents 20 μm. *Di*, quantification of apoptotic cells in total cells in panel (*D*). All experiments were performed in triplicate, and statistical analysis was conducted using Student's *t* test (∗*p* < 0.05, ∗∗*p* < 0.01) or ANOVA (*p* < 0.05). The bars indicate the mean ± SD. DAPI, 4′,6-diamidino-2-phenylindole.

In *H. armigera*, the level of endogenous glucose in hemolymph is about 5‒9 mM during metamorphosis (39). The glucose-mediated autophagic flux was examined by overexpressing the plasmid of red and green fluorescence proteins fused with LC3 (pIEx-4-RFP-GFP-LC3-His) in the *H. armigera* epidermal cell line (HaEpi) (40). After 6 h of treatment, the red and green fluorescence was relatively uniform in the PBS and 5 mM glucose groups; in the 10 mM glucose group, LC3 appeared as autophagic vacuoles exhibiting both RFP and GFP fluorescence, indicating the formation of an autophagosome. In the cells treated for 24 h, LC3 appeared as uniform red and green fluorescence in the PBS group; autophagosomes appeared in the 5 mM glucose group; the green fluorescence was quenched in the 10 mM glucose group, and the acidity of the lysosome quenched the GFP fluorescence, indicating the formation of an autolysosome. However, autophagic vacuoles disappeared after 72 h of glucose treatment, indicating that autophagy did not continuously occur. In the absence of autophagic vacuoles, RFP-GFP-LC3 protein only diffused in the cytoplasm, showing green and red fluorescence (Fig. 6, *A* and *Ai*). After 24 h of induction with 10 mM glucose, LC3-II gradually increased. After 72 h of 5 mM glucose treatment, the amount of LC3-II was significantly higher than that of the control group (Fig. 6, *B* and *Bi*). After 24 h of induction with 10 mM glucose, p62 gradually decreased. After being treated with 5 mM of glucose for 72 h, the protein level of p62 was significantly reduced (Fig. 6, *C* and *Ci*). Furthermore, glucose significantly increased CASP3 activity, as indicated by green fluorescence under 10 mM glucose stimulation for 72 h (Fig. 6, *D* and *Di*). These results suggested that glucose induces autophagy and apoptosis in a time- and concentration-dependent manner.

To determine the role of glucose in lipophagy, we injected glucose into the sixth instar 72 h larvae hemocoel. Glucose injection led to an increase in glucose levels in the hemolymph (Fig. S8*A*). Compared with the control treatment, glucose (200 μg/larva) treatment increased the dissociation of the fat body and LD degradation (Fig. S8*B*). High glucose promoted autophagosome membrane (LC3 indication) sequestration of LDs (Fig. S8, *C* and *Ci*). Glucose treatment increased the colocalization of LDs and lysosomes. The yellow color showed the colocalization of the lysosomes and the LDs (Fig. S8, *D* and *Di*). The TEM showed that, compared with PBS control, the number of LD-containing autolysosomes in the fat body increased after glucose treatment (Fig. S8, *E* and *Ei*). The density of LC3-II was increased in the glucose-injected fat body (Fig. S8*F*). The level of p62 was decreased in the glucose-injected group (Fig. S8*G*). Immunohistochemistry showed that CASP3 was extensively localized in the nuclei in the glucose-injected fat body but in the cytoplasm in the PBS-injected fat body. The TUNEL signal was enhanced by glucose treatment (Fig. S8, *H* and *Hii*). Moreover, the levels of TGs were lower than those in the PBS control (Fig. S8*I*). Glucose treatment promoted the expression of lipase genes and PCD-related genes (Fig. S8*J*). *Atg1* knockdown impeded glucose-induced LC3-II and p62 reduction (Fig. S9, *A* and *B*), and the fusion of lysosomes and LDs (Fig. S9, *C* and *Ci*). D-mannitol was injected as osmotic control under the same conditions as glucose treatment (41). D-mannitol treatment did not alter the density of LC3-II and p62 (Fig. S9, *D* and *E*). Also, D-mannitol treatment did not induce the colocalization of LDs and lysosomes (Fig. S9, *F* and *Fi*), suggesting that changes in osmolarity do not affect lipophagy. These results suggested that high levels of glucose promote lipophagy.

To explore the effect of high glucose levels on lipophagy during metamorphosis, we used metformin hydrochloride (MH) to reduce hemolymph glucose levels. MH (20 μg/larva) did not change glucose levels significantly compared to the control group. Treatment with MH (40–100 μg/larva) significantly reduced hemolymph glucose levels (Fig. S10*A*). MH (40 μg/larva) treatment blocked the dissociation of fat body, and the degradation of LDs (Fig. S10*B*), prevented autophagosome membranes (LC3 indication) from sequestering LDs (Fig. S10, *C* and *Ci*) and the fusion of lysosomes and LDs (Fig. S10, *D* and *Di*), decreased the density of LC3-II (Fig. S10*E*), and prevented p62 degradation (Fig. S10*F*). The signal intensities of CASP3 and TUNEL were decreased after MH injection (Fig. S10, *G* and *Gi*). Compared with the control treatment, MH treatment led to the accumulation of TGs (Fig. S10*H*) and significantly lower mRNA levels of lipase genes and PCD-related genes (Fig. S10, *I* and *J*). These results confirmed the role of glucose in lipophagy.

### High glucose levels supported metamorphosis

Glucose treatment at different concentrations resulted in small pupae of 46 to 52% larvae and the death of 27 to 43% of individuals (Fig. S11, *A* and *B*). The pupation time did not change significantly (Fig. S11*C*), and there was a significant decrease in average pupa weight (Fig. S11*D*). During development, 18 to 24% of pupae failed to emerge, and 51 to 56% of adults were morphologically abnormal (Fig. S11*E*).

When MH was injected into the hemocoel at different concentrations, phenotypes of dead, delayed pupation, and emergence failure were observed (Fig. S11*F*). Treatment with MH (40 μg/larva) resulted in delayed pupation of 51% of larvae and death of 23% of individuals. MH (100 μg/larva) was injected into larvae, pupation was delayed in 21% of larvae, and death occurred in 70% of individuals (Fig. S11*G*). Treatment with MH (40 μg/larva) resulted in an average delay in pupation time of 10 h (Fig. S11*H*), and the average pupa weight did not change (Fig. S11*I*). During development, 74% of pupae had an abnormal phenotype, and 84% of individuals failed to emerge (Fig. S11, *J* and *K*). The expression of *G6pase* and *Pepck* was decreased after MH injection (Fig. S11*L*). These results suggested that hemolymph high glucose levels are essential for metamorphosis.

### High glucose induced FOXO acetylation and nuclear localization to promote gene expression

**Figure 7:**
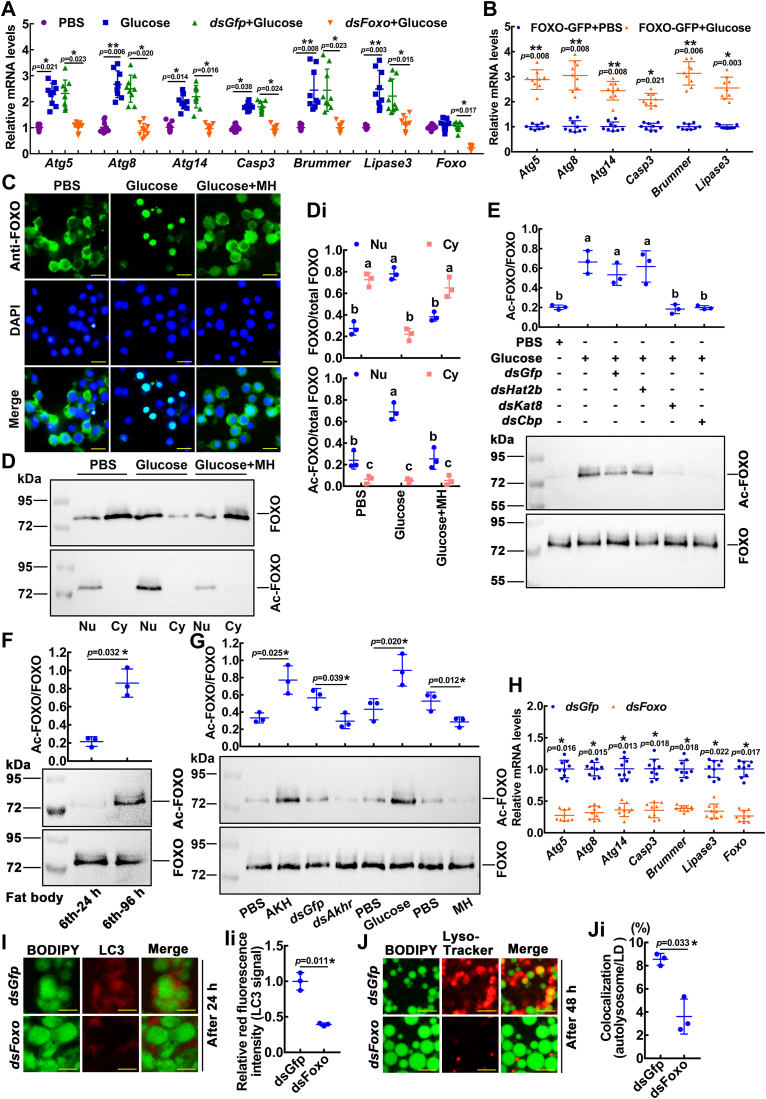
**High glucose promoted the acetylation of FOXO for gene expression.***A*, qRT-PCR showed the mRNA levels of lipase and PCD-related genes after *Foxo* knockdown in HaEpi cells. Subsequently, 48 h after dsRNA (2 μg/well-six well plates) transfection, the cells were treated with glucose. Samples were taken 12 h after glucose treatment. *B*, the mRNA levels of lipase and PCD-related genes after overexpression of FOXO-GFP in HaEpi cells were analyzed by qRT-PCR. Forty-eight hours after FOXO-GFP plasmid (5 μg/well-six well plate) transfection, the cells were treated with glucose. Samples were taken 12 h after glucose treatment. *C*, localization of FOXO in HaEpi cells. *Green* shows that the FOXO protein is stained with an anti-FOXO antibody and a secondary antibody labeled with Alexa Fluor 488. *Blue* shows nuclei stained with DAPI. The ruler represents 20 μm. *D*, Western blotting showed the subcellular distribution and acetylation of FOXO. The gel concentration of SDS‒PAGE was 7.5%. Nu: nucleus, Cy: cytoplasm. *Di*, quantification of FOXO or Ac-FOXO in the nucleus or cytoplasm in total FOXO protein according to three independent replicates using ImageJ software. *E*, Western blotting showed the acetylation of FOXO after *Hat* knockdown. The protein band density was quantified by ImageJ. *F*, the acetylation intensity of FOXO in the fat body at sixth-24 h and sixth-96 h. Western blotting was quantified by ImageJ. *G*, the acetylation intensity of FOXO in the fat body treated with different concentrations of AKH (24 ng/larva), *dsAkhr* (500 ng/larva), glucose (200 μg/larva), and MH (40 μg/larva). Western blotting was quantified by ImageJ. *H*, qRT-PCR showed the mRNA levels of lipase and PCD-related genes after *Foxo* knockdown. Samples were taken 12 h after the third injection. Then, 500 ng dsRNA per sixth-72 h larva was administered three times at a 24 h interval. *I*, the association of LDs with LC3 demonstrated lipophagy processes. Images were collected 24 h after the first injection of dsRNA. The ruler represents 50 μm. *Ii*, statistical analysis of LC3 fluorescence intensity in (*I*). *J*, the association of LDs with lysosomes demonstrated lipophagy processes. Images were collected 48 h after the first injection of dsRNA. The ruler represents 50 μm. *Ji*, statistical analysis of autolysosome numbers in (*J*). The statistical analysis was performed using three independent replicates by Student's *t* test or ANOVA (*p* < 0.05). *Asterisks* denote significant differences (∗*p* < 0.05, ∗∗*p* < 0.01). The bars indicate the mean ± SD. LD, lipid droplet; LC3, microtubule-associated protein 1 light chain 3; FOXO, forkhead box O; MH, metformin hydrochloride; PCD, programmed cell death; qRT-PCR, quantitative real-time reverse transcription PCR; DAPI, 4′,6-diamidino-2-phenylindole.

To demonstrate the mechanism by which glucose promotes lipophagy, we investigated the transcription factor FOXO. Glucose induced upregulation of PCD-related and lipase genes. When *Foxo* expression was decreased, the upregulated expression of glucose-induced PCD-related and lipase genes was inhibited (Fig. 7*A*). Then, FOXO was overexpressed, and PCD-related genes and lipase genes were significantly upregulated under glucose induction (Fig. 7*B*). HaEpi cells were incubated with glucose, and the subcellular localization of FOXO was analyzed by immunocytochemistry. In the PBS-treated control, FOXO was distributed throughout the cell, including the cytoplasm and nucleus. FOXO showed increased nuclear localization after 6 h of incubation with glucose (10 mM); however, MH (1 mM) blocked the glucose-induced nuclear translocation of FOXO (Figs. 7*C* and S12*A*). The acetylation of the FOXO was observed, since the acetylation of FOXO plays an important role in the cell response to stress (42). Western blotting confirmed the increase in acetylated FOXO in the nucleus after glucose incubation. Under MH conditions, FOXO was mainly localized in the cytoplasm (Fig. 7, *D* and *Di*). Using a website (http://csspalm.biocuckoo.org/) to predict FOXO acetylation, we found that four histone acetyltransferases (HATs) may be involved in FOXO acetylation (Fig. S12*B*). We searched for the corresponding HATs in *H. armigera* and conducted knockdown experiments. The acetylation of FOXO induced by high glucose decreased after lysine acetyltransferase 8 (*Kat8*) and CREB-binding protein (*Cbp*) knockdown in the HaEpi cells (Figs. 7*E* and S12, *C*–*E*). The data suggested that high glucose promotes FOXO acetylation through KAT8 and CBP.

FOXO was acetylated during metamorphosis (sixth-96 h) (Fig. 7*F*). AKH treatment increased FOXO acetylation, while *Akhr* knockdown inhibited FOXO acetylation. Glucose treatment increased FOXO acetylation levels, while MH reduced FOXO acetylation (Fig. 7*G*). Knockdown of FOXO during metamorphosis (sixth-72 h) decreased the expression levels of PCD-related genes and lipase genes (Fig. 7*H*). FOXO knockdown prevented autophagosome membrane (LC3 indication) from sequestering LDs and the fusion of lysosomes and LDs (Fig. 7, *I*–*Ji*). Then *Pepck* was knocked down (Fig. S13*A*), resulting in a significant decrease in hemolymph glucose levels (Fig. S13*B*), a decrease in FOXO acetylation (Fig. S13*C* and *Ci*), and the colocalization of lysosomes and LDs were blocked (Fig. S13, *D* and *Di*). *dsPepck* injection resulted in 50% of larvae growing to abnormal pupae, and 77.8% of pupae growing to abnormal adults (Fig. S13, *E*–*G*), suggesting that endogenous glucose leads to acetylation of FOXO and lipophagy, thus promoting metamorphosis. These results revealed that high glucose increases FOXO acetylation and induces FOXO nuclear localization *in vivo* to promote the related gene expression for lipophagy.

## Discussion

The regulatory mechanism of glycolipid metabolism is extremely intricate. The mechanism by which the glucagon-like hormone AKH promotes lipophagy has not been elucidated, and the relationship between the steroid hormone 20E and peptide hormone AKH has yet to be demonstrated. The present study revealed that 20E promotes gluconeogenesis and increases hemolymph glucose levels by promoting the expression of *Akh* and *Akhr*. High glucose levels induced the acetylation of FOXO for the expression of PCD-related genes and lipase genes, and promoted lipophagy and apoptosis.

### Lipophagy is necessary for insect metamorphosis

Autophagy plays a crucial role in regulating the homeostasis of liver lipid metabolism. Enhanced levels of autophagy lead to liver fat consumption and reduce liver lipid accumulation (43). Inhibition of autophagy causes lipid accumulation in the liver, leading to severe disease (44). Similarly, we found that reduce autophagy levels led to the accumulation of TGs in the fat body of *H. armigera*. Lipophagy, in which autophagosomes selectively engulf LDs for lysosomal degradation, is essential for lipid and energy homeostasis. Lack of lipophagy is associated with metabolic disorders, such as liver steatosis (45, 46). The lipophagy receptor ORP8 KO, the mouse shows defective lipid clearance in the liver (18). Our study proved that lipophagy plays an essential role in the metamorphosis and development of insects, and blocking lipophagy leads to abnormal pupae and emergence failure, which hinders the normal life process of insects.

### 20E promotes *Akh* and *Akhr* transcription *via* EcR

GCs promote glucagon level increase by enhancement of α-cell secretory function in rats (24). Glucagon is derived from the cleavage of proglucagon, and proglucagon is encoded by the preproglucagon gene (*Gcg*). Specific transcription factors (Isl-1, Pax, Cdx, and so on) and distinct DNA control elements (G1, G2, G3, G4, and so on) in the preproglucagon promoter region initiate or inhibit preproglucagon expression (47, 48). The action of glucagon is mediated through interaction with a specific GCGR. The transcription factor specificity protein 3 (SP3) is found to promote the expression of the GCGR gene in the mouse pancreatic MIN6 β-cell line (49). There are no studies to clarify the mechanism by which steroid hormones regulate the transcription of glucagon and GCGRs. In *Locusta migratoria*, the adipokinetic hormone/corazonin-related peptide precursor gene displays strong transcriptional responses to prolonged flight (50). In *Drosophila melanogaster*, unknown regulatory factor(s) act on the *Akh* promoter to regulate the *Akh* gene (51). Similarly, upregulation of *Akh* and *Akhr* at the transcriptional level in response to 20E occurred. However, the transcriptional regulatory mechanism of *Akh* and *Akhr* by steroid hormones has not been reported. Our results indicated that 20E upregulates *Akh* and *Akhr* expression in insects *via* EcR. The study reveals the mechanism by which steroid hormones promote glucagon-like peptide hormone expression through transcriptional regulation.

### AKH pathway increases hemolymph glucose levels by promoting gluconeogenic gene expression to promote lipophagy

Glucagon increases glucose levels by promoting glycogen breakdown and gluconeogenesis (52). Glucagon promotes the expression of *G6Pase* and *Pepck* through FOXO, cAMP response element binding protein (CREB), and some nuclear receptors (53). AKH is the insect analog of glucagon, and AKH injection induces glycogen mobilization (54). The present study further clarifies that AKH increases hemolymph glucose levels by increasing the expression of the gluconeogenic genes *G6pase* and *Pepck*. Our study provides a supplement to the mechanism by which AKH elevates hemolymph glucose.

AKH promotes lipolysis by promoting the high expression of lipase through its receptor AKHR (54). In addition to the roles of the cytoplasmic lipases, it is now demonstrated that autophagy is involved in mobilizing LDs during periods of nutrient stress (55, 56). The increase in basal autophagy levels leads to an increase in autophagic vesicles, and the increase in contact between autophagic vesicles and LDs leads to lipophagy (55, 57). Lipophagy is one kind of selective macroautophagy. Macroautophagy is classified as selective or nonselective. Nonselective macroautophagy is the random isolation of cytoplasmic material by autophagosomes, whereas selective macroautophagy causes the lysosomal degradation of specific cargoes, and the pathways are named according to the type of cargo, including LDs (lipophagy), aggregated proteins (aggrephagy), peroxisomes (pexophagy), and mitochondria (mitophagy) (58, 59). LDs are conserved cellular organelles that store neutral lipids and fulfill critical functions in lipid and energy homeostasis (60). Lipophagy involves the autophagosome-mediated sequestration of LDs and their subsequent delivery to lysosomes for the turnover of LDs to free fatty acids (57, 61). 20E induces autophagy in the *Drosophila* fat body (62). In *B. mori*, 20E upregulates *Atg* genes to induce autophagy in the fat body (36). 20E upregulates the expression of the transcription factor Krüppel-like factor 15 (*Klf15*) to promote macroautophagy/autophagy and apoptosis in the *H. armigera* fat body (35). However, during the metamorphosis of insects, the mechanism of lipophagy is unknown. Our study revealed that the elevation of glucose triggered by 20E promotes lipophagy.

### High glucose levels induce FOXO acetylation for lipophagy-related gene expression

Glucose presents energy for body growth, however, high glucose promotes autophagy and apoptosis of MC3T3-E1 osteoblasts (63) and *D. melanogaster* retinal neurons (64). In people with type two diabetes, high glucose can lead to an increase in FOXO-1 acetylation, triggering autophagy and apoptosis and leading to tissue damage (65, 66). High glucose significantly increases FOXO-1 acetylation in mouse microvascular endothelial cells, which can be reduced by metformin (MH) (41), a widely used drug to decrease human plasma glucose levels (67). FOXO1 acetylation induces the expression of autophagy and autophagy inducer genes in human colon cancer cell HCT116 (68). In *H. armigera*, 20E induces nonphosphorylated FOXO to enter the nucleus to increase the expression of the transcription factor *Brz7* (69). In the present study, high glucose levels promoted FOXO acetylation and nuclear translocation to induce the expression of PCD-related genes and lipase genes. The HAT CBP enhances FOXO1 acetylation by interacting with FOXO1 in HEK293T cells (70). In our study, RNA interference experiments confirmed that KAT8 and CBP are involved in glucose-induced FOXO acetylation. Still, the mechanism of FOXO acetylation and FOXO acetylation sites remains to be further explored. These studies suggest that the mechanism by which high glucose-induced acetylation of FOXO promotes gene transcription is evolutionarily conserved.

## Conclusions

**Figure 8:**
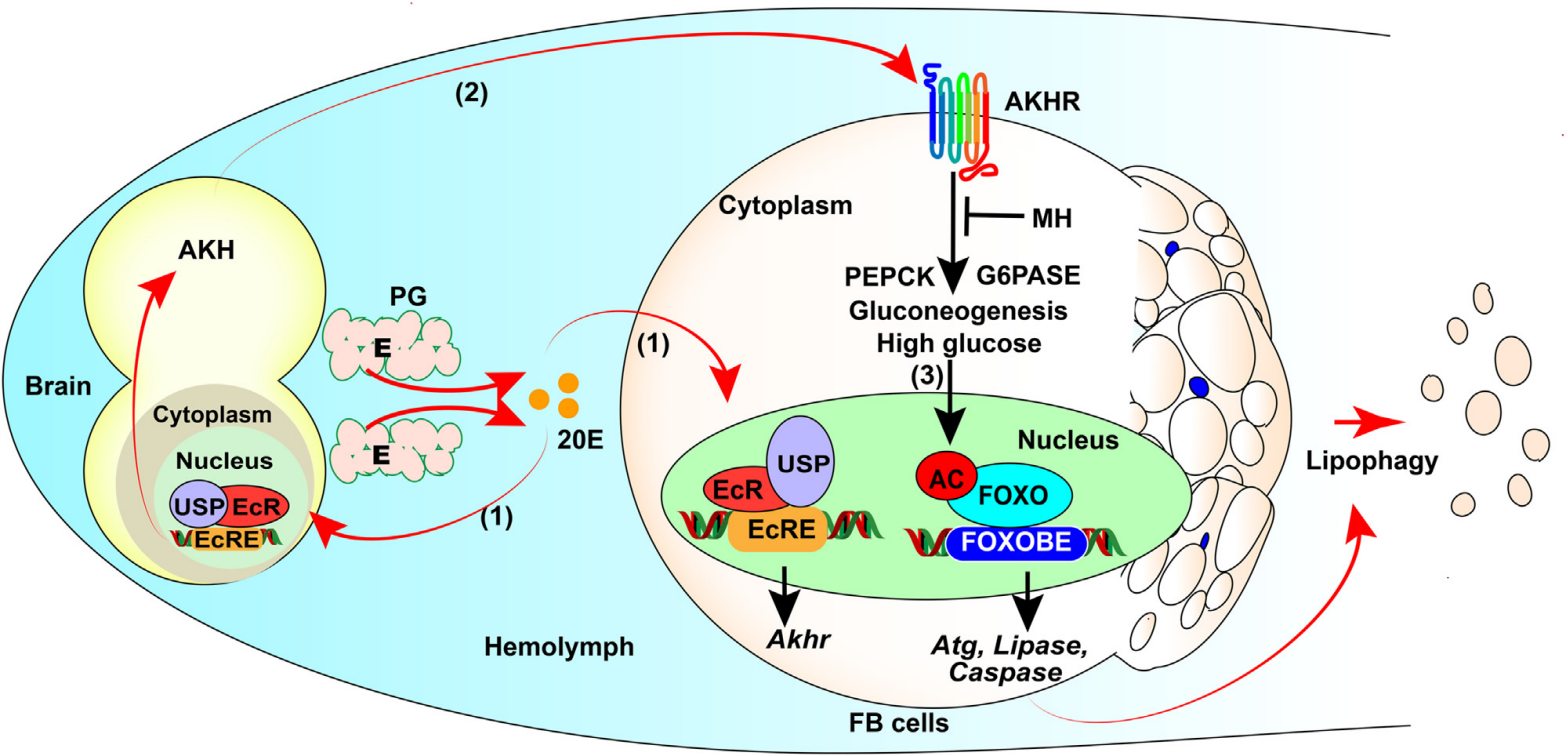
**Chart illustrating the mechanisms by which the steroid hormone 20E promotes lipophagy by elevating glucose levels.** 20E promotes the transcription of *Akh* and *Akhr via* EcR (1). AKH *via* AKHR to increase the expression of gluconeogenesis-related genes to increase glucose levels (2). High glucose induces FOXO acetylation and nuclear localization, promotes gene expression, and then promotes lipophagy and apoptosis (3). 20E, 20-hydroxyecdysone; AKH, adipokinetic hormone; AKHR, AKH receptor; FOXO, forkhead box O.

20E promotes gluconeogenesis and increases hemolymph glucose levels by promoting the expression of glucagon-like hormone AKH and AKHR. The high levels of glucose present energy for the development after feeding stop and induce the acetylation of FOXO for the expression of PCD-related genes and lipase genes to induce lipophagy of the fat body during insect metamorphosis. Lipophagy is essential for lipid metabolism in insects and ensures various life activities during metamorphosis without feeding (Fig. 8).

## Experimental procedures

### Insects

*H. armigera* was maintained in our laboratory at 26 ± 1 °C under light with a 14 h light/10 h dark cycle and 60 to 70% relative humidity. The larvae were reared on the artificial diet described in previous work (71).

### Cell culture

The *H. armigera* epidermal cell line (HaEpi) was established in our laboratory and has been well characterized previously (40). HaEpi cells developed as a loosely attached monolayer and were maintained at 27 °C with Grace's insect cell culture medium (Gibco) containing 10% fetal bovine serum (Biological Industries).

### Whole-mount immunohistochemistry

After being rinsed in PBS, the fat body was fixed in 4% paraformaldehyde for 2 h at room temperature. The samples were blocked with 5% bovine serum albumin (BSA, PBS with 0.3% Triton X-100) for 3 h and then incubated with a primary antibody against LC3 (Abcam) at 4 °C for 24 h. DyLight 594-conjugated goat anti-rabbit immunoglobulin G (IgG) (Abbkine) was used as the secondary antibody and incubated at room temperature in the dark for 2 h. The samples were rinsed with PBS. The tissues were incubated with 5 μM BODIPY dye (Macklin) for 30 min at 37 °C in darkness and rinsed two times in PBS. Fluorescence was detected using an Olympus BX51 fluorescence microscope (Olympus Optical Co).

### Lyso-Tracker and BODIPY staining

The fat body tissues were rinsed three times in PBS. At room temperature and away from light, the fat body tissues were incubated with 5 μM BODIPY dye (Macklin) for 30 min and rinsed two times in PBS. Then, 50 nM LysoTracker (Thermo Fisher Scientific) was incubated with the fat body for 10 min in 37 °C darkness, and the fat body was laid on a glass slide and covered with a cover slide. Fluorescence microscopy (Olympus Optical Co) was used for observation and imaging.

### Double-stranded RNA synthesis

The effect of RNAi has been tested in a variety of moths (72). When long dsRNA is broken down into smaller fragments *in vivo*, it specifically inhibits the expression of target genes in worms (73, 74). RNAi primers containing the T7 sequence (Table S1) were used to obtain the DNA template by PCR. For the dsRNA transcription system, 2 μg of DNA template, 20 μl of 5 × transcription buffer, 3 μl of T7RNA polymerase (20 U/μl), 2.4 μl of A/U/C/GTP (10 mM) each, 3 μl of RNase inhibitor (40 U/μl, Thermo Fisher Scientific), and RNase-free water were mixed to a volume of 50 μl. After incubation at 37 °C for 4 to 6 h, 10 μl of RNase-free DNase I (1 U/μl, Thermo Fisher Scientific), 10 μl of DNase I buffer, and 30 μl of RNase-free water were added to the solution, which was incubated at 37 °C for 1 h. The product was purified with phenol/chloroform and precipitated with ethanol; the precipitate was resuspended in 50 μl of RNase-free water. The purity and integrity of the dsRNA were determined using agarose gel electrophoresis. A MicroSpectrophotometer (GeneQuant; Amersham Biosciences) was used to quantify the dsRNAs.

### RNAi of genes in larvae

The larvae were placed on ice for 15 min to make them immobile. The dsRNA was diluted to 100 ng/μl with PBS. A sterile microsyringe was used to inject 5 μl of diluted dsRNA into the larval hemocoel at 24 h intervals. *dsGfp* was used as a control. Total RNA or protein was extracted to detect the effects of RNAi at 24 h after the last injection. When 80% of the individuals in the control group were pupated, the phenotype ratio was counted, and phenotype pictures were taken. Individuals with large differences were excluded, 30 individuals were used for statistical analysis, and three independent replicates were performed.

### Quantitative real-time reverse transcription PCR

Total RNA was extracted by TRIzol reagent (TransGen Biotech), and first-strand complementary DNA was synthesized using 5 × All-In-One RT Master Mix (abm) according to the manufacturer's instructions. Quantitative real-time reverse transcription PCR was carried out using the qTOWER^3^/G system (Analytik Jena AG) with TransStart Tip Green qPCR Supermix (AiDLab) and the primers listed in Table S1. The relative expression levels of the genes were quantified using *Actb* (β-actin) expression as the internal control. The data were analyzed by the 2^−ΔΔCT^ method (ΔΔCT = ΔCT_treated sample_ − ΔCT_control_, ΔCT = CT_gene_ − CT_Actb_) (75).

### Induction with 20E in larvae

The 20E powder was dissolved into a 20 mM storage solution with DMSO. The 20E was diluted in PBS to the corresponding concentration and injected into the hemocoel of sixth instar 6 h larvae. DMSO was diluted equally as a control.

### Luciferase reporter assay

NCBI was used to find the promoter sequences of *Akh* and *Akhr*, and the target fragment was inserted into pIEx-4-luciferase-GFP-His (LUC-GFP-His) to construct *pAkh*/*pAkhr*-LUC-GFP-His. Transcription factor-binding sites were predicted according to the JASPAR website (https://jaspar.genereg.net/). EcR-RFP-His was cotransfected with *pAkh*/*pAkhr*-LUC-GFP-His. After 24 h of cotransfection, cells were incubated with 20E (2 μM) or DMSO for 48 h. The expression of LUC-GFP-His was detected by Western blotting. EcR-RFP-His, pRL-TK, and *pAkh*/*pAkhr*-LUC-GFP-His were cotransfected, and after 24 h, the cells were incubated with 20E or DMSO for 24 h. Luciferase activity was measured using a Dualucif Firefly and Renilla Assay Kit (UElandy) according to the instructions.

### Western blotting

The larval tissues were dissected from the body along the midline and rinsed the epidermis, midgut, and fat body separately in PBS, placed the tissues in 500 μl Tris–HCl buffer (pH 7.5, 40 mM) with 5 μl PMSF on ice and ground them fully. The samples were centrifuged at 10,000*g* for 15 min, and the supernatant was retained and mixed in protein loading buffer, then incubated in a 100 °C water bath for 15 min. The cells in the six-well plate were rinsed with PBS, the cell suspension was collected, centrifuged at 1000*g* for 5 min, the precipitation was collected, and the PBS suspension precipitation and mixed with the protein loading buffer, and incubated in a 100 °C water bath for 15 min.

Total proteins were extracted from cells or larvae. An equal amount of each sample (∼20 μg) was subjected to 7.5%-15% SDS‒PAGE and then transferred onto a nitrocellulose membrane by electrotransfer. The membranes were blocked with a blocking buffer containing 5% nonfat milk in Tris-buffered saline (TBS; 10 mM Tris–HCl, 150 mM NaCl, pH 7.5) for 1 h at room temperature. Then, the membranes were incubated in a blocking buffer with primary antibody (Anti-ORP8 antibody, 1:2000; Anti-GFP/RFP/ACTB antibody, 1:5000; Anti-LC3 antibody, 1:1000; Anti-FOXO antibody, 1:1000; anti-acetyl Lysine antibody, 1:2000; Anti-IDE, 1:1000; Anti-p62, 1:1000) at 4 °C overnight. The membranes were washed with TBST (0.02% Tween in TBS) for 10 min each, the membranes were incubated with secondary antibody (Goat Anti-Rabbit/-Mouse IgG-HRP conjugate secondary antibody, 1:5000 diluted secondary antibody) for 2 h at room temperature. The membranes were washed with TBST and then one wash for 5 min with TBS, the membrane was covered with High-sigECL Western Blotting Substrate (Tanon Science & Technology). The chemiluminescent signal was detected using a 5200 Chemiluminescence Imaging System (Tanon Science & Technology).

### ChIP assay

For transcription factor-binding sites, the EcR-RFP-His plasmids were transfected into HaEpi cells for 72 h (cells were approximately 70% confluent). The cells were then treated with 2 μM 20E for 6 h to detect the binding of EcR in the promoters of *Akh* and *Akhr*. DMSO treatment was used as a control. The ChIP Assay Kit (P2078, Beyotime Biotechnology) was used for the following assay according to the instructions. Anti-His antibody was used to immunoprecipitate EcR-RFP-His, and IgG was used as a negative control.

### *In situ* hybridization

The brain tissue (sixth-24 h and sixth-96 h) was quickly dissected, rinsed with PBS, and fixed in 4% paraformaldehyde at room temperature for 2 h. The sample was digested with protease K (20 μg/ml) at 37 °C for 30 min. The sample was rinsed once with distilled water. Tissue samples were incubated with a prehybridization solution without a probe at 37 °C for 3 h. The prehybridization solution was discarded, and the oligonucleotide probe (500 ng/ml) labeled with digoxin was added overnight at 37 °C. The sample was rinsed twice with saline sodium citrate buffer (2 × SSC, 300 mM NaCl, and 30 mM C_6_H_5_O_7_Na_3_·2H_2_O) for 5 min each time. 0.5 × SSC washed samples for 15 min; 0.2 × SSC washed samples for 15 min. The sample was incubated with a blocking buffer at 37 °C for 30 min. The biotinylated anti-digoxin antibody was incubated samples at 37 °C for 60 min. The samples are washed with 0.5 M PBS. The avidin-FITC was added to samples at 37 °C for 30 min. 0.5 M PBS washed samples. The slides were sealed with a mounting medium and observed under a confocal microscope (Leica). Digoxin-labeled RNA probes were synthesized by Sangon Biotech (Sangon Biotech). The kit was from Boster Biological Technology (Boster).

### AKH treatment

AKH peptides (Pyr-EITFSRDWTG-NH_2_) were synthesized by a biological company (Sangon Biotech). AKH powder was dissolved in 40 mM storage solution with DMSO and diluted to the corresponding concentration with PBS (140 mM NaCl, 10 mM sodium phosphate, pH 7.4). At the sixth instar 72 h, larvae were injected with AKH at 24 h intervals for a total of three times, with PBS as a control.

### H&E staining

The fat body tissue was isolated, rinsed with PBS, and fixed in 4% paraformaldehyde at 4 °C overnight. The fixed tissues were dehydrated gradually using different buffers. The tissues were embedded in paraffin, and after the paraffin was allowed to solidify, 7 μm sections were sliced using a paraffin-slicing machine. The sections were flattened in a 60 °C water bath and immediately adhered to a glass slide. The slides were dried at 60 °C for 6 h and subsequently dewaxed. The tissue sections were stained using a H&E) Stain Kit (Solarbio) according to the manufacturer's instructions. The images were observed using an Olympus BX51 fluorescence microscope (Olympus Optical Co).

### Nile red staining

The fat body tissue was obtained from the dissected body quickly, rinsed with PBS, wiped with filter paper, kept the tissue flat, dropped the embedding agent on the surrounding area, and frozen until the embedding agent and the tissue frozen into white ice body. Then the slice was prepared. The frozen sections were left at room temperature for 20 min and allowed to dry. The slides were immersed in 4% paraformaldehyde solution and fixed at room temperature for 30 min. The sections were washed with PBS twice. The liquid around the sample was blotted with filter paper. The tissue was covered with Nile red (MCE) solution and incubated at 37 °C for 1 h in the dark. The dye solution was removed and washed with PBS in the dark three times. The 4′,6-diamidino-2-phenylindole (DAPI) (BBI LIFE SCIENCE) solution was incubated in the dark for 10 min. The dye solution was removed and washed with PBS in the dark three times. An antifluorescence quencher was added to the fixed tissue slides, and the slides were sealed for observation.

### TUNEL staining

The fat body tissue was rinsed with PBS and fixed overnight with 4% paraformaldehyde at 4 °C. Then, gradually dehydrated with different buffers. The tissue was embedded in paraffin, and the paraffin sections were prepared after the paraffin solidified. The fat body tissue paraffin section was removed, and the liquid around the section sample was carefully blotted with filter paper. The sample area was covered with proteinase K solution (20 μg/ml) and incubated for 20 min at room temperature. The sections were washed with PBS 2 times for 5 min each time, and the excess liquid was removed by suction with filter paper. The TUNEL reaction was performed according to the instructions of the TUNEL Apoptosis kit (UElandy). The red fluorescence intensity (tunnel signal) was observed using an Olympus BX51 fluorescence microscope (Olympus Optical Co).

### Determination of TG levels

After treatment of the larvae, fat body tissues were collected, and tissues were rinsed with PBS. A total of 0.1 g of fat body tissue was placed into a 1.5 ml centrifuge tube, and the solution provided by the kit was added for ice bath homogenization. The samples were centrifuged at 8000*g* for 10 min at 4 °C, and the supernatant was collected and analyzed according to the instructions of the TG content detection kit (Solarbio). The color development intensity of the TG reaction was measured by spectrophotometry at 420 nm (Infinite M200PRO NanoQuant).

### Hemolymph glucose level determination

Hemolymph (30 μl) was collected from at least three larvae into centrifuge tube containing 1.5 μl of 1 mM N-phenylthiourea and centrifuged at 3000*g* for 5 min to remove blood cells. Subsequently, 10 μl of the supernatant was used for hemolymph glucose determination according to the instructions of the glucose assay kit (Mlbio). The intensity of the color was determined at 505 nm by spectrophotometry (Infinite M200PRO NanoQuant).

### Glucose treatment

Glucose powder (Sinopharm) was dissolved in sterile water and configured as a 1 M glucose storage solution. Sterile PBS was used to prepare the corresponding concentration of glucose diluent. At sixth instar 72 h, larvae were injected with glucose (200 μg/larva, 400 μg/larva, and 800 μg/larva) at 24 h intervals, with PBS as a control. When incubating cells with glucose, the corresponding glucose solution (5 mM and 10 mM) was added according to the volume of the cell medium.

### MH treatment

Metformin hydrochloride powder (Solarbio) was prepared into a 20 mg/ml solution with sterilized water. MH (20 μg/larva, 40 μg/larva, and 100 μg/larva) was injected into larvae at sixth instar 72 h using sterile PBS configuration to the corresponding concentration. PBS was used as a solvent control.

### Immunocytochemistry

HaEpi cells were seeded at a density of 70% on cover glass slides in 24-well cell culture plates. The cells were incubated with different media containing PBS, 10 mM glucose alone, or with MH (1 mM) for 12 h. The cells were fixed in 4% paraformaldehyde in the dark for 10 min and washed three times with PBS. The cells were blocked with 2% BSA in PBS for 1 h. Then, the cells were incubated with rabbit polyclonal antibodies against FOXO as the primary antibody (1:200 dilution in 2% BSA) overnight at 4 °C. The cells were washed and subsequently incubated with goat anti-rabbit IgG Alexa Fluor 488 (diluted 1:1000 in 2% BSA) for 1 h at 37 °C. The nuclei were stained with DAPI for 10 min at room temperature. The negative control was treated following the same method but with the primary antibody replaced with preserum. Fluorescence was detected using an Olympus BX51 fluorescence microscope (Olympus Optical Co).

### Autophagy detection

The LC3 antibody was purchased from Abcam company (ab109364, Abcam). The green fluorescent protein is quenched at acidic pH, and GFP is fused with LC3 to produce GFP-LC3, which indicates the development of autophagy from autophagosomes (neutral pH) to autophagic solution (acidic pH) according to the change in green fluorescence (76). The pIEx-4-RFP-GFP-LC3-His reporter plasmid was overexpressed for 48 h in HaEpi cells to detect autophagosomes and autolysosomes (77). Autophagic flux was shown by counting the number of autophagosome and autolysosome puncta in successfully transfected pIEx-4-RFP-GFP-LC3-His cells, and images are from the results of three experiments.

### CASP3 activity assay in HaEpi

In glucose-treated HaEpi cells, the activity of CASP3 was detected using a SuperView 488 caspase 3 assay kit (UElandy) *via* immunocytochemistry, according to the manufacturer's instructions. SuperView 488 Caspase-3 Substrate (5 μM) was added to the cell culture media, followed by a 30 min incubation at room temperature. The nuclei were stained with 10 μg/ml DAPI (BBI LIFE SCIENCES) for 10 min, and the signals were observed with an Olympus BX51 fluorescence microscope (Olympus Optical Co).

### FOXO acetylation assay

The cells were lysed by 500 μl of radioimmunoprecipitation assay lysis buffer for 20 min and harvested by centrifugation at 100,00*g* for 15 min at 4 °C. The fat body tissue was collected after different treatment. The fat body was completely grinded with 500 μl of radioimmunoprecipitation assay lysis buffer, the homogenate was centrifuged at 10,000*g* at 4 °C for 15 min, and then the supernatant was collected. The supernatant and 50 μl of protein A were shaken for 1 h at 4 °C to reduce nonspecific binding. The supernatant was collected by centrifugation. Anti-FOXO antibody and supernatant were mixed and incubated overnight at 4 °C. Then 50 μl of protein A was added to samples, the mixture was shaken for 4 h and washed three times with lysis buffer. The precipitate was resuspended with 50 μl of lysis buffer. The samples were used for Western blotting. FOXO was used as a protein quality and quantity control. FOXO acetylation was detected with anti-acetyl lysine antibody.

### Bioinformatic analysis

The active peptide of *H. armigera* AKH was identified and compared with AKH of different species by DNAMAN software (http://www.lynnon.com/) (Fig. S14*A*). The phylogenetic tree was constructed using the neighbor-joining method in MEGA 7.0 (Fig. S14*B*).

### The antibodies used in this study

Polyclonal anti-ORP8 antibody (anti-OSBPL8 antibody) was obtained from Boster Biological Technology. Monoclonal anti-GFP antibody, monoclonal anti-RFP antibody, and polyclonal anti-ACTB antibody were obtained from ABclonal Technology (ABclonal). The LC3 antibody was purchased from Abcam company (ab109364, Abcam). The rabbit anti-acetyl Lysine antibody was obtained from ImmunoChem (ImmuneChem) The rabbit polyclonal antibody against *H. armigera* CASP3 (anti-CASP3 antibody) was prepared in our laboratory (78). Polyclonal anti-FOXO was prepared in our laboratory using recombinantly expressed protein in *Escherichia coli*. Polyclonal anti-p62 was prepared in our laboratory using recombinantly expressed protein in *E. coli*. The specificity of the antibody was detected by Western blotting (Fig. S15).

### Ethics statement

The antibody preparation in rabbit was in accordance with protocols approved by the Animal Care and Welfare Committee at Shandong University School of Life Sciences (SYDWLL-2021–54).

### Statistical analysis

All data were obtained from at least three biological replicates. The density of protein bands of Western blotting was analyzed using ImageJ software (National Institutes of Health, http://imagej.nih.gov/ij/download.html). We applied Excel and GraphPad 7 software (GraphPad, Inc., https://www.graphpad.com/) to analyze the data and generate the figures. Student's *t* test (two-tailed) was used to analyze two-group datasets. An asterisk represented a significant difference (*∗p* < 0.05), and two asterisks represent extremely significant differences (*∗∗p* < 0.01) in the figures. The bars indicated the mean ± SD of three biological replicates. Multiple sets of data were compared by one-way analysis of variance (ANOVA). The different lowercase letters showed significant differences.

## Data availability

All data are contained within the article. This article contains supporting information.

## Supporting information

This article contains supporting information.

## Conflict of interest

The authors declare that they have no conflicts of interest with the contents of this article.
