# Supplementary material for: The steroid hormone 20-hydroxyecdysone induces lipophagy via the brain-adipose tissue axis by promoting the adipokinetic hormone pathway
Source: J Biol Chem. 2025 Jan 10;301(2):108179. doi: 10.1016/j.jbc.2025.108179 (PMC11835591; doi:10.1016/j.jbc.2025.108179)
Supplement: Supporting information [file mmc1.docx]

**The steroid hormone 20-hydroxyecdysone induces lipophagy via the brain-****adipose tissue axis by promoting the adipokinetic hormone pathway**

Yan-Xue Li^1^, Yan-Li Li^1^, Xiao-Pei Wang^1^, Tian-Wen Liu^1^, Du-Juan Dong^1^, Jin-Xing Wang^1^ and Xiao-Fan Zhao^1^[[1]](#footnote-1)^*^

^1^ Shandong Provincial Key Laboratory of Animal Cells and Developmental Biology, School of Life Sciences, Shandong University, China

**Supporting information**

Supplemental Figures and legends: Figure S1-S15

Supplemental Table: Table S1

**
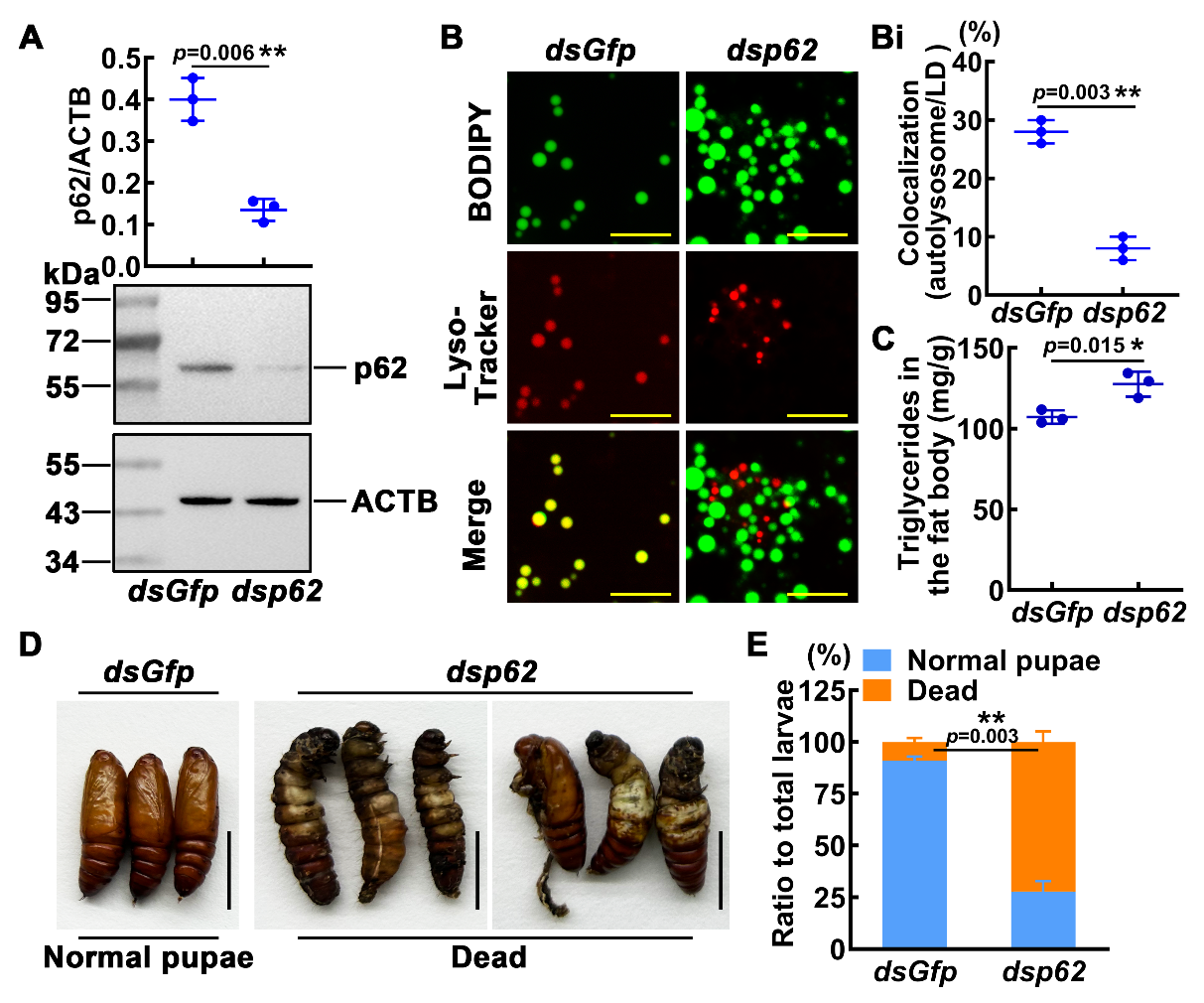
**

**Figure S1. Knockdown of *p62* blocked lipophagy.** *A,* The expression of p62 was analyzed by western blotting. (6th-6 h with first injection, once every 24 h, a total of four injections). Samples were taken 144 h after the first injection of dsRNA. *B* and *Bi,* The colocalization of LDs and lysosomes was detected after *p62* knockdown. The ruler represents 50 μm. Images were collected 144 after the first injection of dsRNA. *C,* The levels of triglycerides in the fat body after *p62* knockdown. *D,* Phenotypes after *dsp62* injection. Bars = 1 cm. *dsGfp* was used as a control. *E,* Statistical analysis of the phenotypes in (D). The bars indicate the mean ± SD. *p* values and asterisks indicate differences by two-tailed Student's *t* test (**p* < 0.05, ***p* < 0.01).

**
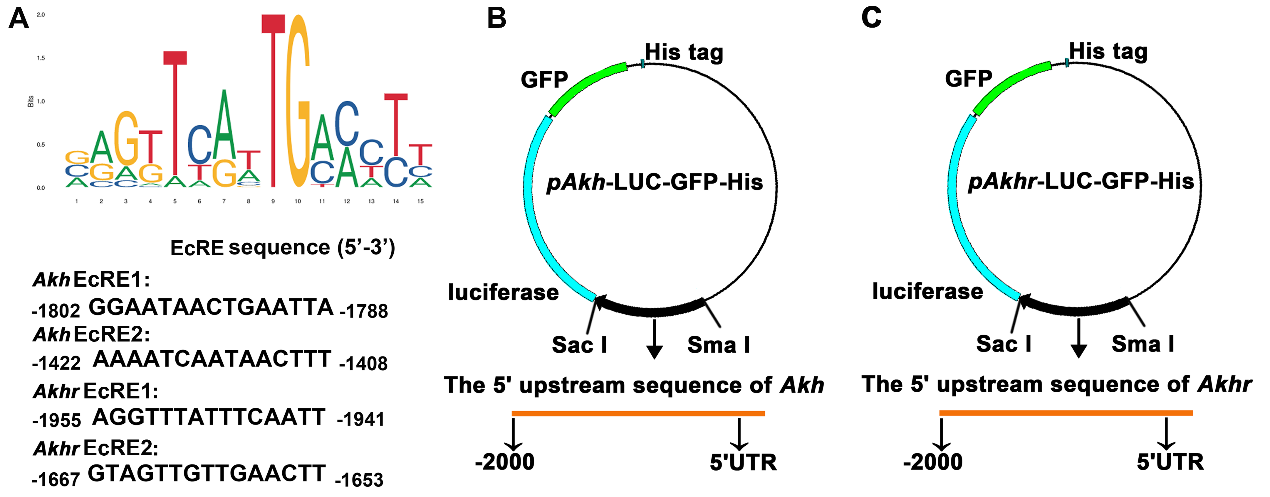
**

**Figure S2. The map of report plasmid.** *A,* Alignment of the EcRE sites in the promoter of *Akh* and *Akhr* predicted by the JASPAR transcription factor database. *B* and *C,* The report plasmid schematic of *Akh* and *Akhr*.

**
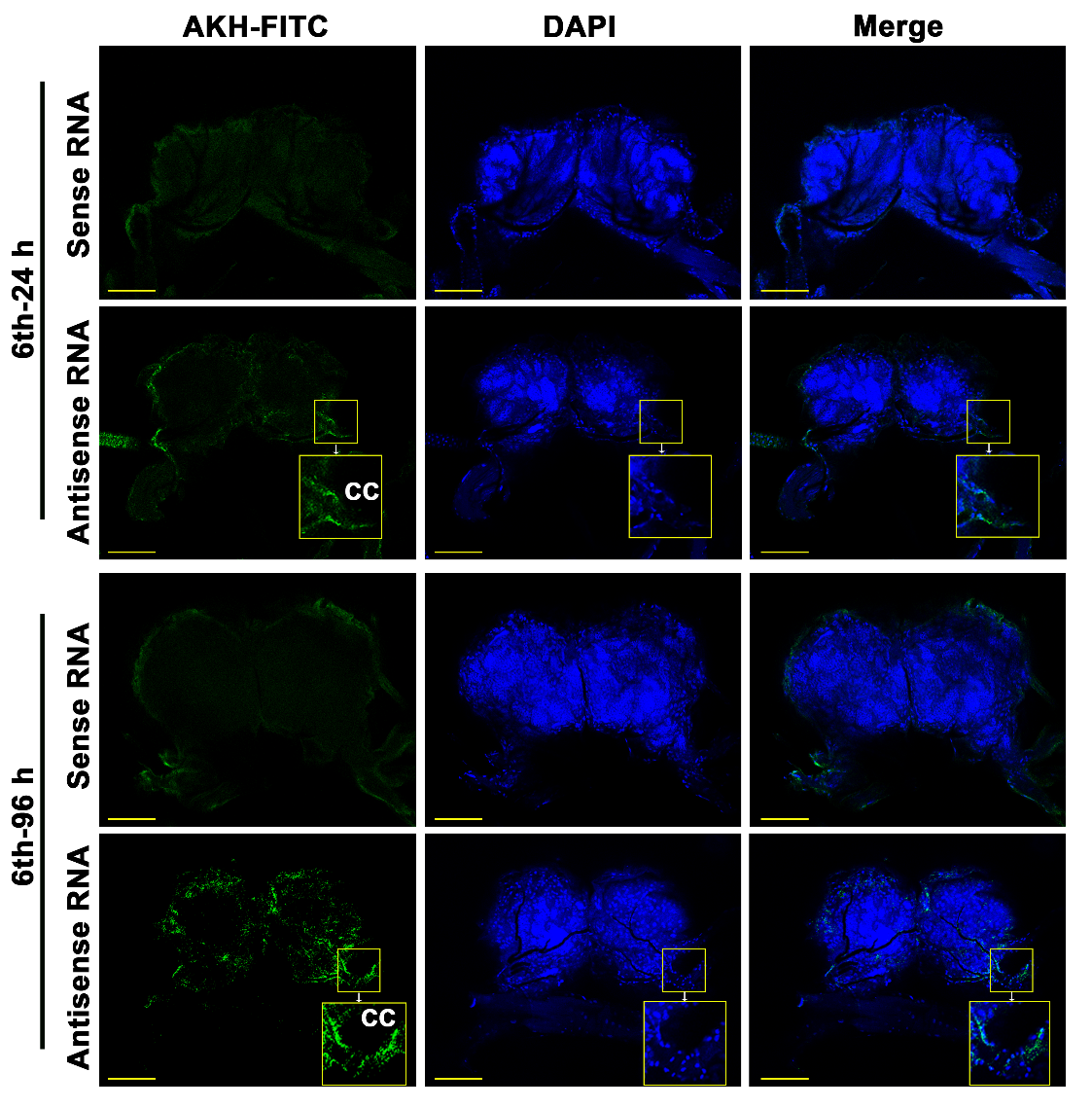
**

**Figure S3. The localization of AKH was analyzed by in situ hybridization.** Green fluorescence represented AKH. DAPI: nuclear staining. 6th-24 h: sixth instar 24 h larvae; 6th-96 h: sixth instar 96 h larvae. The ruler represents 100 μm. CC: corpora cardiaca.


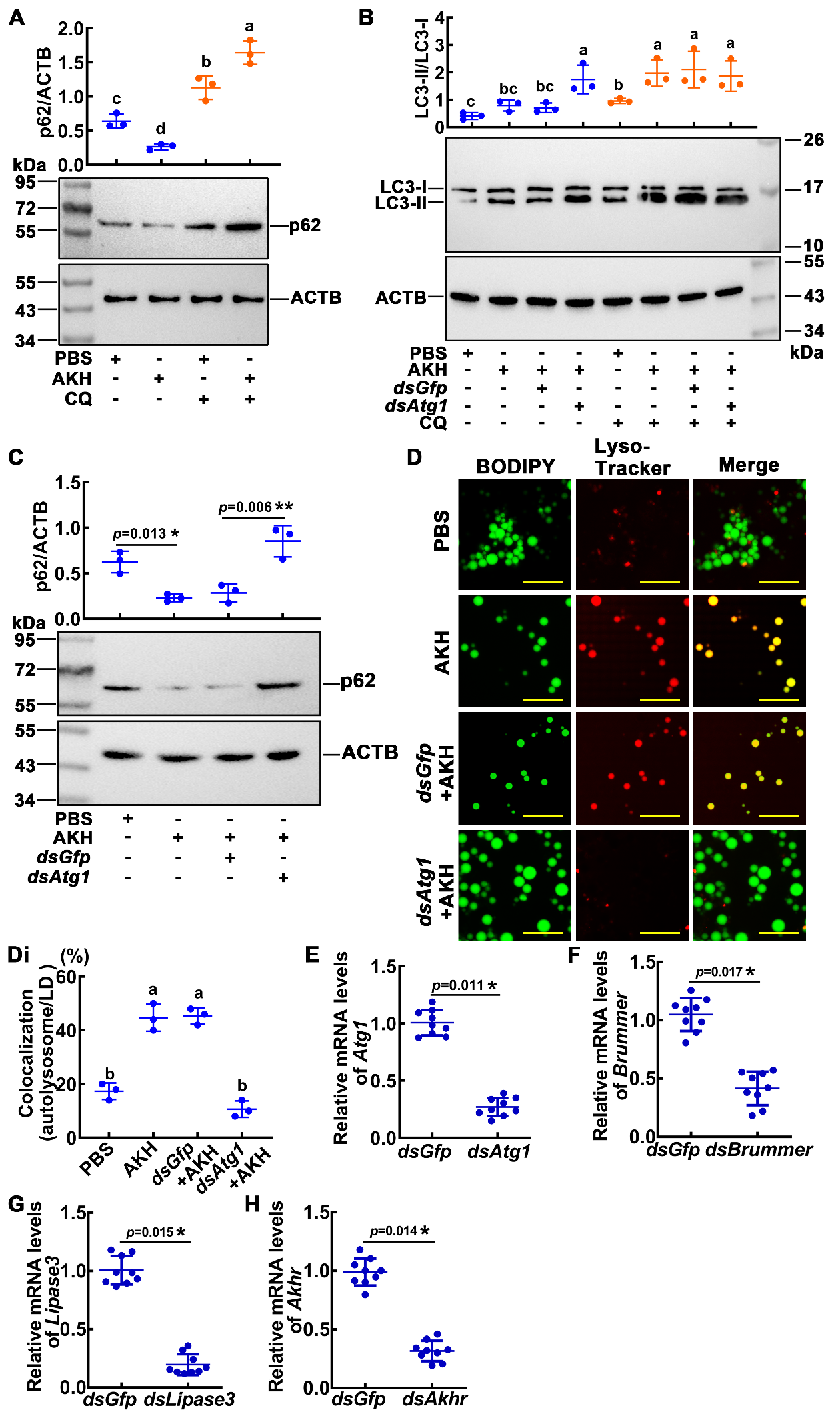


**Figure S4. Knockdown of *Atg1* blocked AKH-induced lipophagy.** *A,* Western blotting detection of p62 protein levels in the fat body. CQ: chloroquine, 10 μM for 24 h. The samples were obtained 48 h after the first injection of AKH. *B,* LC3-II levels were visualized by western blotting with antibodies against LC3. Samples were taken 144 h after the first injection of dsRNA. AKH treatment for 48 h. CQ: chloroquine, 10 μM for 24 h. *C,* p62 levels were visualized by western blotting with antibodies against p62. Samples were taken 144 h after the first injection of dsRNA. AKH treatment for 48 h. *D,* Colocalization of LDs and lysosomes. BODIPY (5 μM for 30 min, green fluorescence). Lyso-Tracker (50 nM for 10 min, red fluorescence). Merge was the overlap of red and green fluorescence. Scale bars = 50 µm. Images were collected 144 h after the first injection of dsRNA. *Di,* The ratio of lipophagy LDs (yellow) to total LDs (green). *E,* The mRNA levels of *Atg1* after dsRNA injection were analyzed by qPCR. *F,* RNAi efficiency of *Brummer* in the fat body*. G,* Knockdown efficiency of *lipase3* in the fat body*.* *H,* RNAi efficiency of *Akhr* in the fat body*.* Data were mean ± SD of three replicates. The statistical analysis was performed using three independent replicates by ANOVA or Student's *t* test.


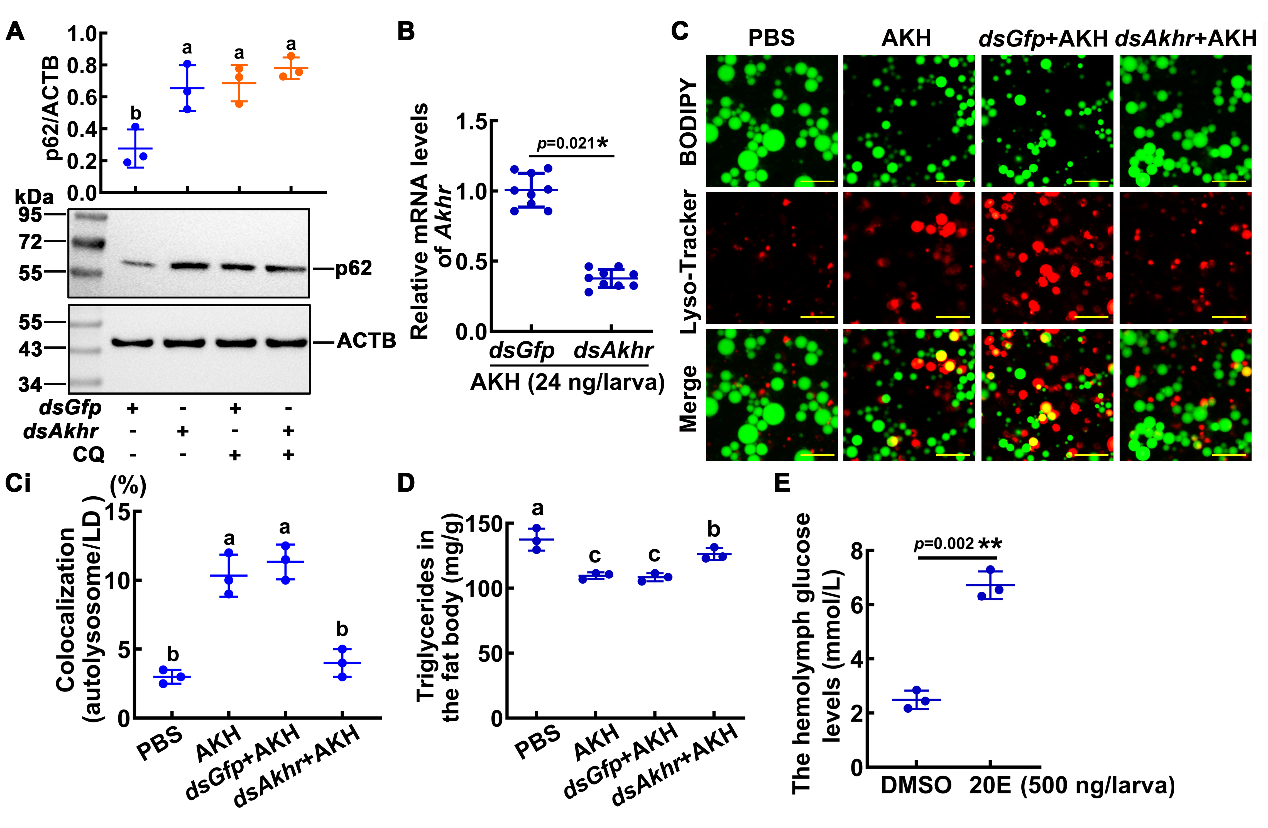


**Figure S5. *Akhr* knockdown repressed AKH-induced lipophagy.** *A,* p62 levels were visualized by western blotting with antibodies against p62. CQ: chloroquine, 10 μM for 24 h. Samples were taken 144 h after the first injection of dsRNA. *B,* The mRNA levels after dsRNA injection were analyzed by qPCR. dsRNA injection at 6th-6 h, thrice at a 24 h interval. The AKH injection for 24 h after the last dsRNA injection. *C,* Colocalization of LDs and lysosomes. BODIPY (5 μM for 30 min, green fluorescence). Lyso-Tracker (50 nM for 10 min, red fluorescence). Merge was the overlap of red and green fluorescence. Scale bars = 50 µm. *Ci,* The ratio of lipophagy LDs (yellow) to total LDs (green). *D,* Measurement of triglyceride levels in the fat body. The samples were obtained 24 h after the AKH injection. *E,* The increase of the hemolymph glucose levels by 20E induction. The sixth instar 6 h larvae was injected 20E for 12 h. DMSO was used as the solvent control. The bars indicate the mean ± SD of three times repetition. The statistical analysis was performed using three independent replicates by ANOVA. **p* < 0.05, ***p* < 0.01 by two-tailed Student's *t* test.


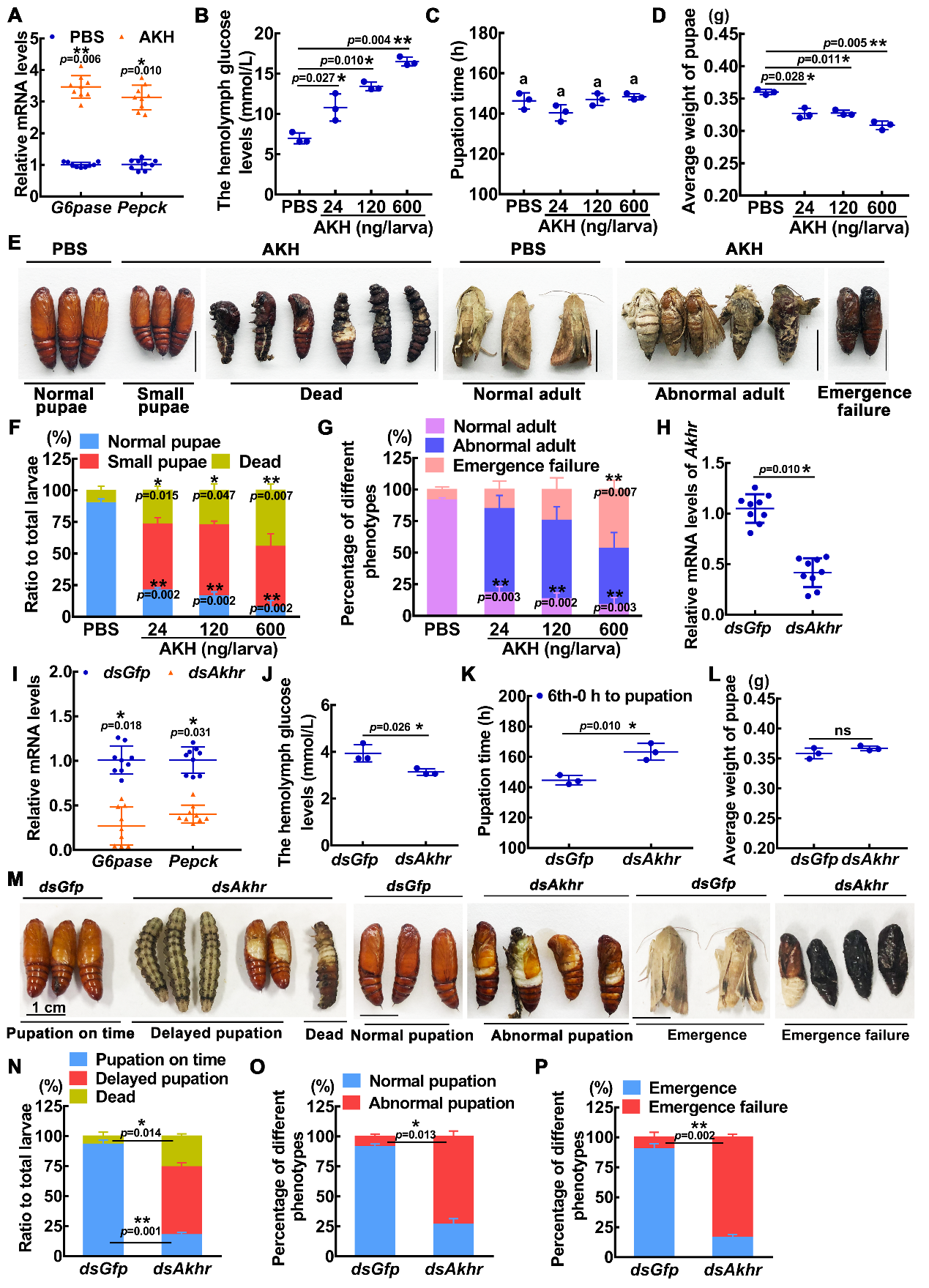


**Figure S6. The AKH pathway elevated glucose levels to promote metamorphosis.** *A,* The mRNA levels of *G6pase* and *Pepck* after the first AKH (24 ng/larva, once every 24 h at larva of 6th-72 h, with a total of three injections) injection for 48 h, analyzed by qRT‒PCR. *B,* The hemolymph glucose levels after AKH injection. *C,* Statistical analysis of pupation time from 6th instar 0 h larvae to pupae. *D,* Statistical analysis of the average weight of pupae. *E,* Phenotypes after AKH injection. Bars = 1 cm. PBS was used as a control. *F,* Statistical analysis of the phenotypes in (E). *G,* Percentage of different phenotypes from pupae to adults. *H,* The efficiency of *Akhr* knockdown (dsRNA 500 ng per 6th-6 h larva, a total of four injections at a 24 h interval). *I,* qRT‒PCR analysis of the mRNA levels of *G6pase* and *Pepck* after knockdown of *Akhr*. *J,* The hemolymph glucose levels after dsRNA injection. *K,* Statistical analysis of pupation time from sixth instar 0 h larvae to pupae. *L,* Statistical analysis of the average weight of pupae. *M,* Phenotypes after knockdown of *Akhr*. The bars represent 1 cm. *N*-*P,* Percentage of different phenotypes from pupae to adults. The bars indicate the mean ± SD. *p* values and asterisks indicate differences by two-tailed Student's *t* test (**p* < 0.05, ***p* < 0.01).

**
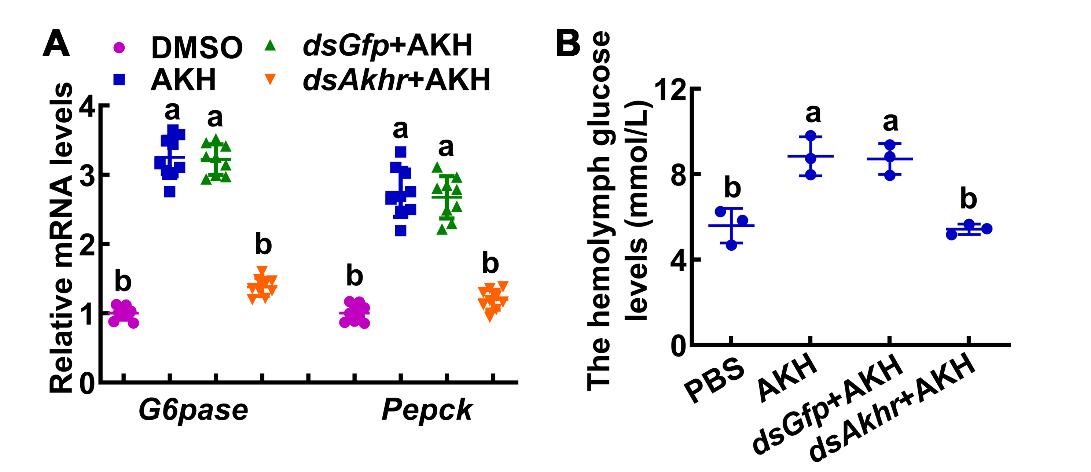
**

**Figure S7. *Akhr* knockdown repressed AKH-induced gene expression.** *A,* The mRNA levels of *G6pase* and *Pepck* were analyzed by qRT‒PCR (AKH 24 ng/larva, for 24 h). *B,* The hemolymph glucose levels were measured after different treatments. The samples were obtained after the AKH injection for 24 h. The statistical analysis was performed using three independent replicates by ANOVA.


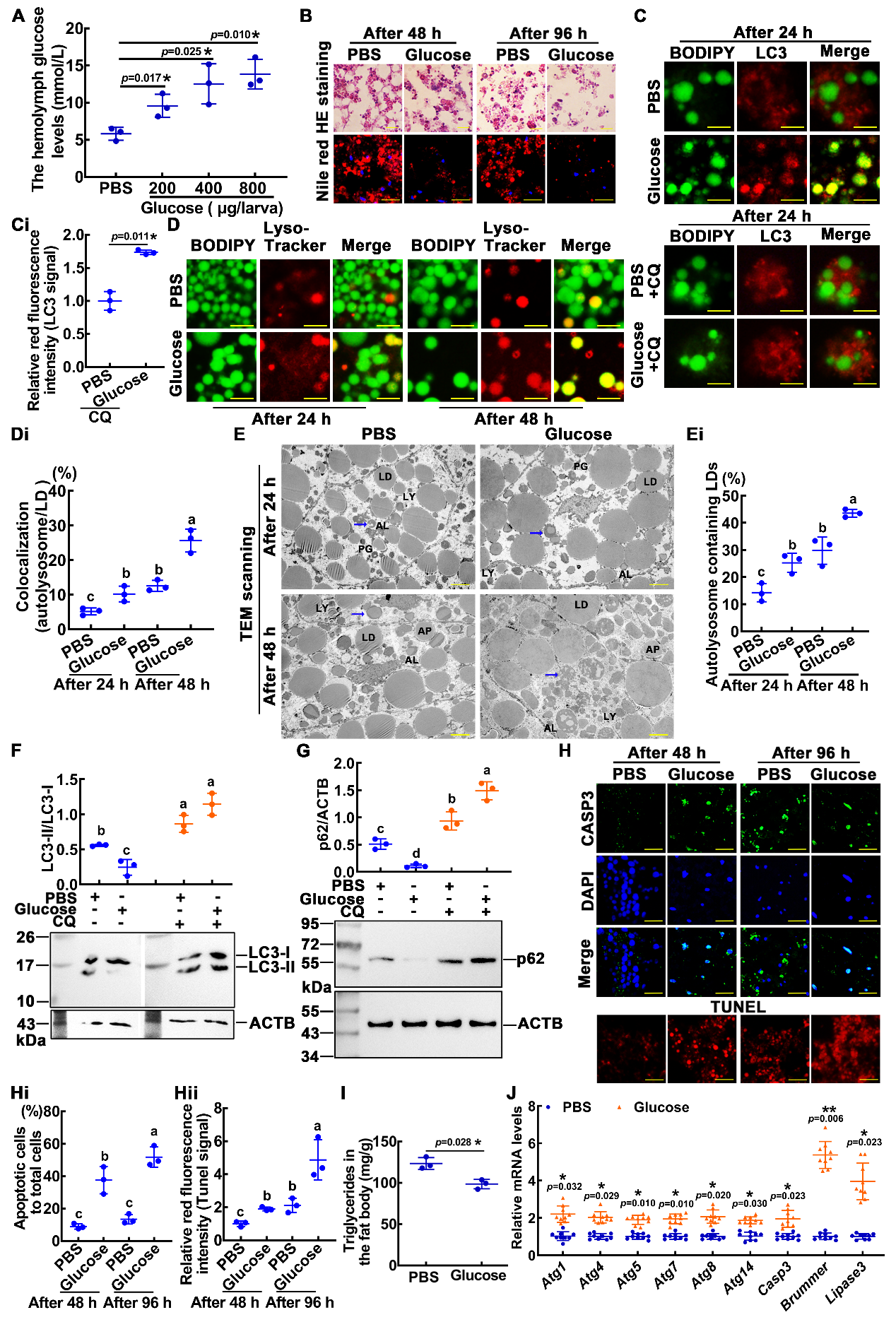


**Figure S8.** **Injection of glucose promoted lipophagy in the fat body.** *A,* The hemolymph glucose levels were measured after the injection of different concentrations of glucose. The samples were obtained after the second glucose injection (once every 24 h at larva of 6th-72 h, with three injections) for 12 h. Glucose: 200 μg/larva = 5 mM. *B,* HE staining and Nile red staining showed the morphology of the fat body after glucose (200 μg/larva, once every 24 h at 6th-72 h, with a total of three injections) injection. The scale bar is 20 µm in HE staining and 50 µm in Nile red staining. Glucose: 200 μg/larva = 5 mM. *C,* The colocalization of LC3 (red) and LDs (green). The ruler represents 50 μm. ImageJ software was used to transform the image data. *Ci,* Statistical analysis of the LC3 signal intensity. *D,* The colocalization of lysosomes (red) and LDs (green) in fat body cells. Yellow indicates lipophagy. The ruler represents 50 μm. *Di,* The ratio of lipophagy LDs (yellow) to total LDs (green). *E,* TEM observation after injection with glucose in the fat body. Blue arrows indicate that LD is entrapped in the lysosome. The bars represent 50 μm. LD: lipid droplet; PG: phagophore; AP: autophagosome; LY: lysosome; AL: autolysosome. *Ei,* The ratio of autolysosome containing LDs to total LDs. *F,* Western blotting detection of LC3-II protein levels in the fat body. CQ: chloroquine, 10 μM for 24 h. The samples were obtained 48 h after the first injection. *G,* Western blotting detection of p62 protein levels in the fat body. CQ: chloroquine, 10 μM for 24 h. The samples were obtained 48 h after the first injection of glucose. *H,* CASP3 location in the fat body and TUNEL signal. Green fluorescence: CASP3 location. Blue fluorescence: DAPI staining nuclei. Red fluorescence: apoptotic signals. The bars represent 50 μm. *Hi,* Statistical analysis of apoptotic cells in (H). *Hii,* Statistical analysis of tunel fluorescence signal intensity in (H). *I,* Triglyceride levels were measured after glucose injection, with PBS as a control. The samples were obtained 48 h after the first injection. *J,* The expression levels of lipase and PCD-related genes were analyzed by qRT‒PCR. Statistical analysis was conducted using ANOVA (*p* < 0.05) or Student's *t* test (**p* < 0.05; ***p* < 0.01). The bars indicate the mean ± SD of three replicates.

**
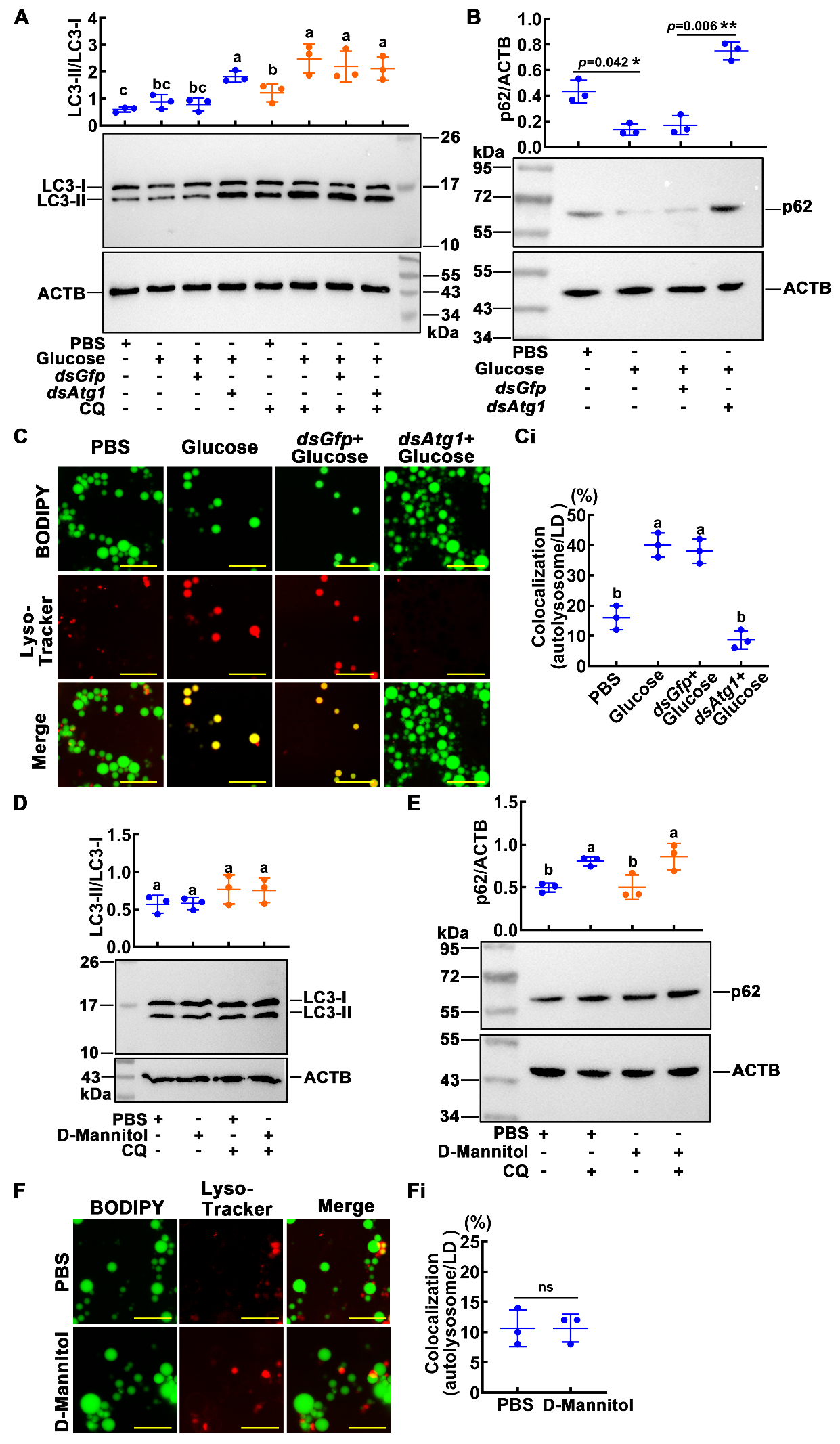
**

**Figure S9. Knockdown of *Atg1* blocked glucose-induced lipophagy and D-mannitol did not affect lipophagy.** *A,* Western blotting detection of LC3-II levels in the fat body. Samples were taken 144 h after the first injection of dsRNA. Glucose treatment for 48 h. CQ: chloroquine, 10 μM for 24 h. *B,* Western blotting detection of p62 protein levels in the fat body. Samples were taken 144 h after the first injection of dsRNA. Glucose treatment for 48 h. *C,* The colocalization of lysosomes and LDs in fat body cells. Yellow indicates lipophagy. The ruler represents 50 μm. Images were collected 144 h after the first injection of dsRNA. *Ci,* The ratio of lipophagy LDs (yellow) to total LDs (green). *D,* Effect of D-mannitol injection on LC3-II in the fat body. The D-mannitol (200 ug/larva) was injected larva at 6th-72 with 24 h interval, LC3-II protein levels were determined by western blotting. Fat body samples were taken 48 h after the first injection. The protein band density was quantified by ImageJ. The gel concentration of SDS-PAGE was 15%. *E,* Effect of D-mannitol injection on p62 levels in the fat body. The gel concentration of SDS-PAGE was 10%. *F,* The colocalization of lysosomes and LDs in fat body cells. Yellow indicates lipophagy. The ruler represents 50 μm. *Fi,* The ratio of lipophagy LDs (yellow) to total LDs (green). Statistical analysis was conducted using ANOVA (*p* < 0.05) or Student's *t* test (**p* < 0.05; ***p* < 0.01). The bars indicate the mean ± SD of three replicates.


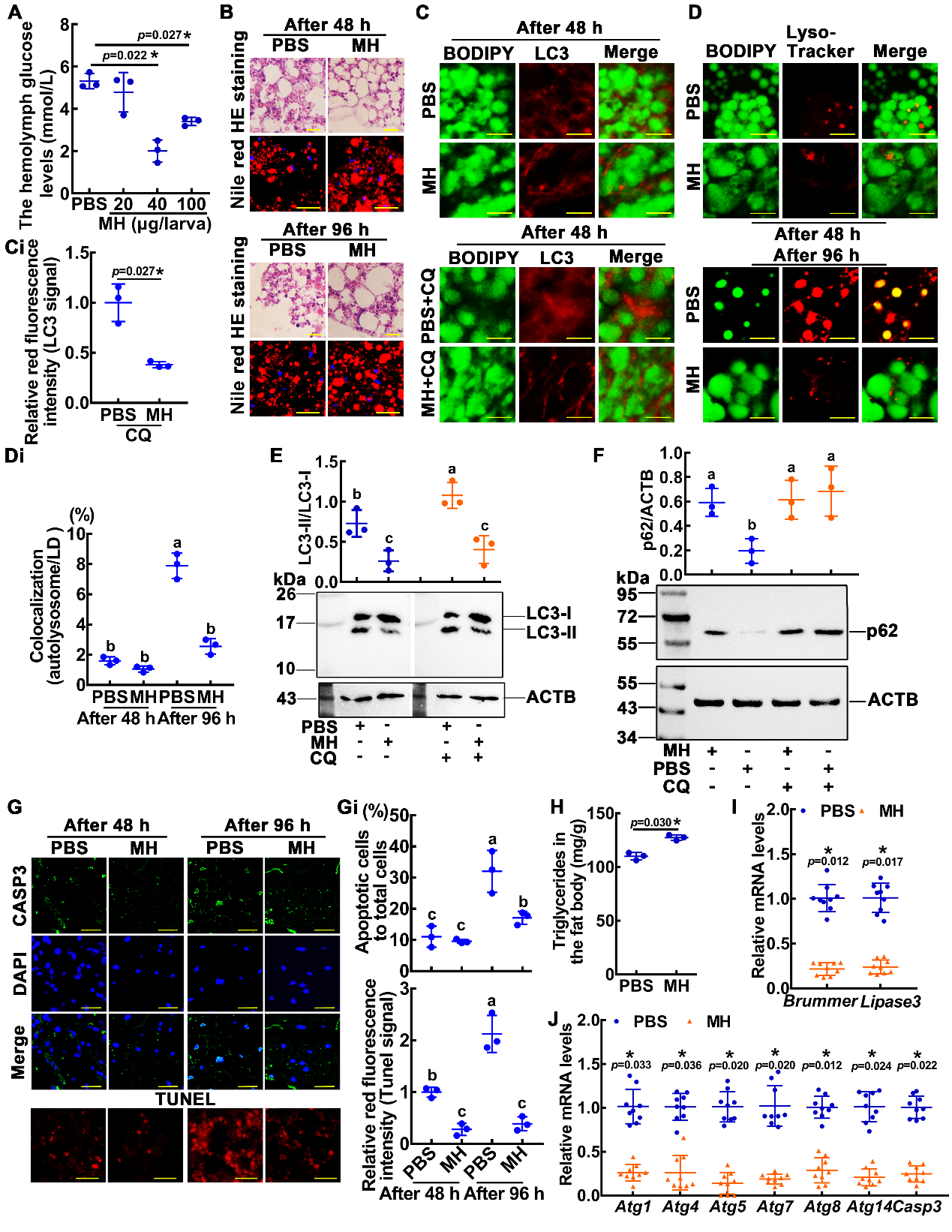


**Figure S10.** **MH injection inhibited lipophagy and apoptosis.** *A,* The hemolymph glucose levels were measured after the injection of different concentrations of MH. The samples were obtained after the second MH injection (once every 24 h at larva of 6th-72 h, with a total of three injections) for 12 h. *B,* HE- and Nile red-stained fat body after MH (40 μg/larva at 6th-72 h, thrice at a 24 h interval) treatment. PBS was used as a control. LDs were marked in red, and nuclei were marked in blue. HE images scale bar: 20 µm, Nile red image scale bar: 50 µm. MH: 40 μg/larva = 1.2 mM. *C,* The fat body was stained with BODIPY to visualize LDs, and immunohistochemistry was performed with an antibody against LC3 (red) to show LC3 protein. The ruler represents 50 μm. *Ci,* Statistical analysis of LC3 fluorescence intensity in (C). *D,* The colocalization of lysosomes and LDs in fat body cells. Lysosomes were stained with Lyso-Tracker (red), and LDs were stained with BODIPY (green). Yellow indicates examples of Lyso-Tracker-positive structures containing LDs. The ruler represents 50 μm. *Di,* Statistical analysis of autolysosome numbers in (D). *E,* Western blotting detection of LC3-II protein levels in the fat body. ACTB was used as a protein loading control, 15% SDS‒PAGE gel. *F,* Western blotting detection of p62 protein levels in the fat body. ACTB was used as a protein loading control, 10% SDS‒PAGE gel. *G,* Detection of cell apoptosis by CASP3 and TUNEL. Green fluorescence indicates CASP3. Blue fluorescence: DAPI staining nuclei. Red fluorescence represents the TUNEL signal. The bars represent 50 μm. *Gi,* Statistical analysis of CASP3 nuclear localization and TUNEL signal in (G). *H,* The levels of triglycerides in the fat body. *I,* The expression level of the lipase gene was analyzed by qRT‒PCR. *J,* Expression levels of PCD-related genes after MH treatment. All experiments were performed in triplicate, and statistical analysis was conducted using ANOVA (*p* < 0.05) or Student's *t* test (**p* < 0.05; ***p* < 0.01). The bars indicate the mean ± SD.

**
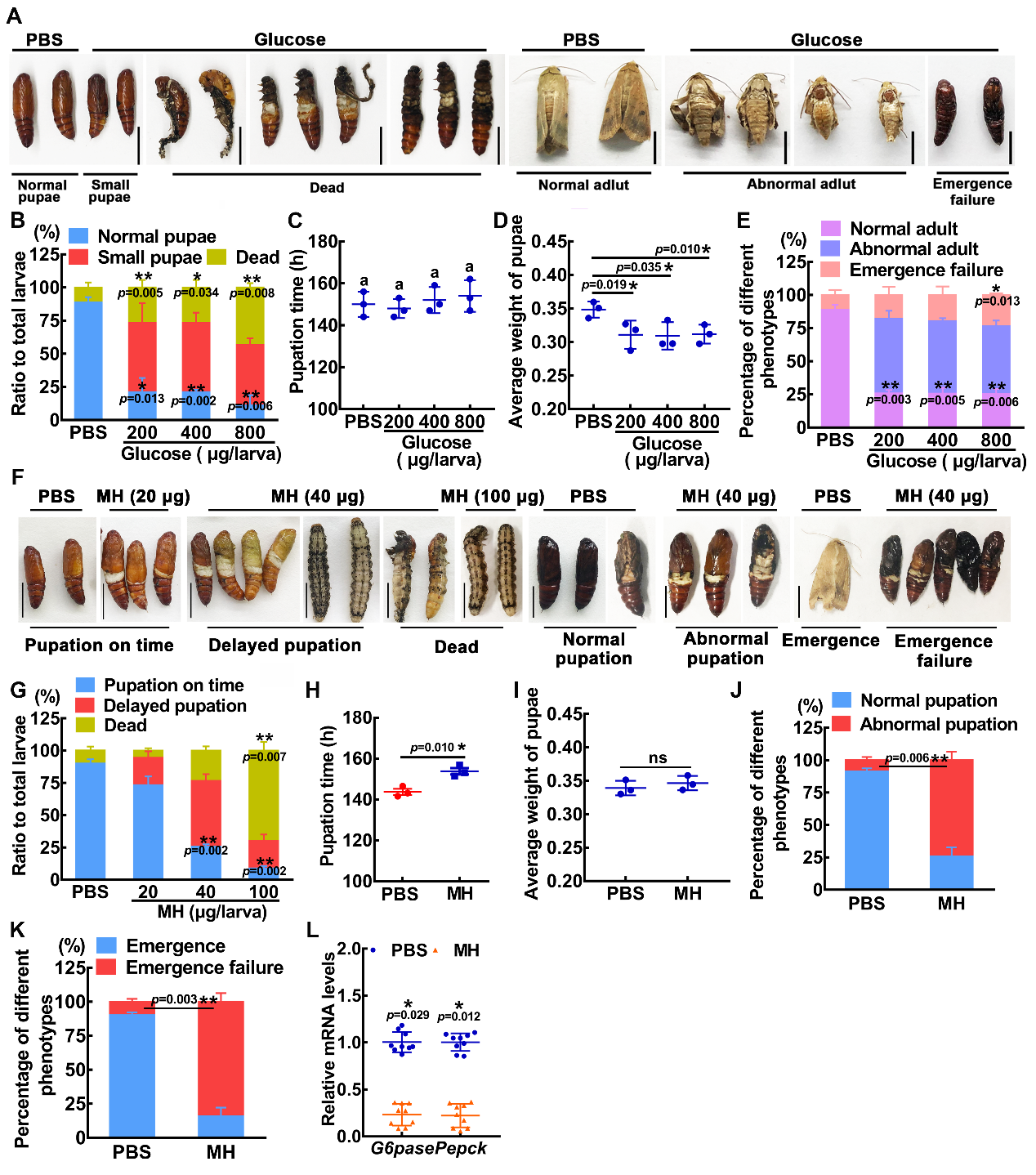
**

**Figure S11. Injection of glucose or MH resulted in metamorphosis failure.** *A,* Phenotypes after glucose injection into hemocoel at larva of 6th-72 h (once every 24 h, with a total of three injections). The ruler represents 1 cm. PBS was used as the control. Glucose: 200 μg/larva = 5 mM. *B,* Ratio of phenotypes at pupation stage. *C,* The time at which the larvae pupated after glucose injection. *D,* The average weight of pupae after glucose injection. *E,* The ratio of phenotypes of adults to all survival pupae. *F,* Phenotypes after MH injection (at 6th-72 h, thrice at a 24 h interval). MH: 20 μg/larva = 0.6 mM. *G,* Statistical analysis of the pupal phenotype in (F). *H,* The pupation time from 6th instar 0 h to pupa 0 d under MH (40 μg/larva) treatment. *I,* The average weight of pupae after MH injection. *J* and *K,* Statistical analysis of the emergence phenotype in (F) under MH (40 μg/larva) treatment. *L,* The mRNA levels of *G6pase* and *Pepck* after MH injection. Data were mean ± SD of three replicates. **p* < 0.05, ***p* < 0.01 (two-tailed Student's *t* test). The different lowercase letters show significant differences.


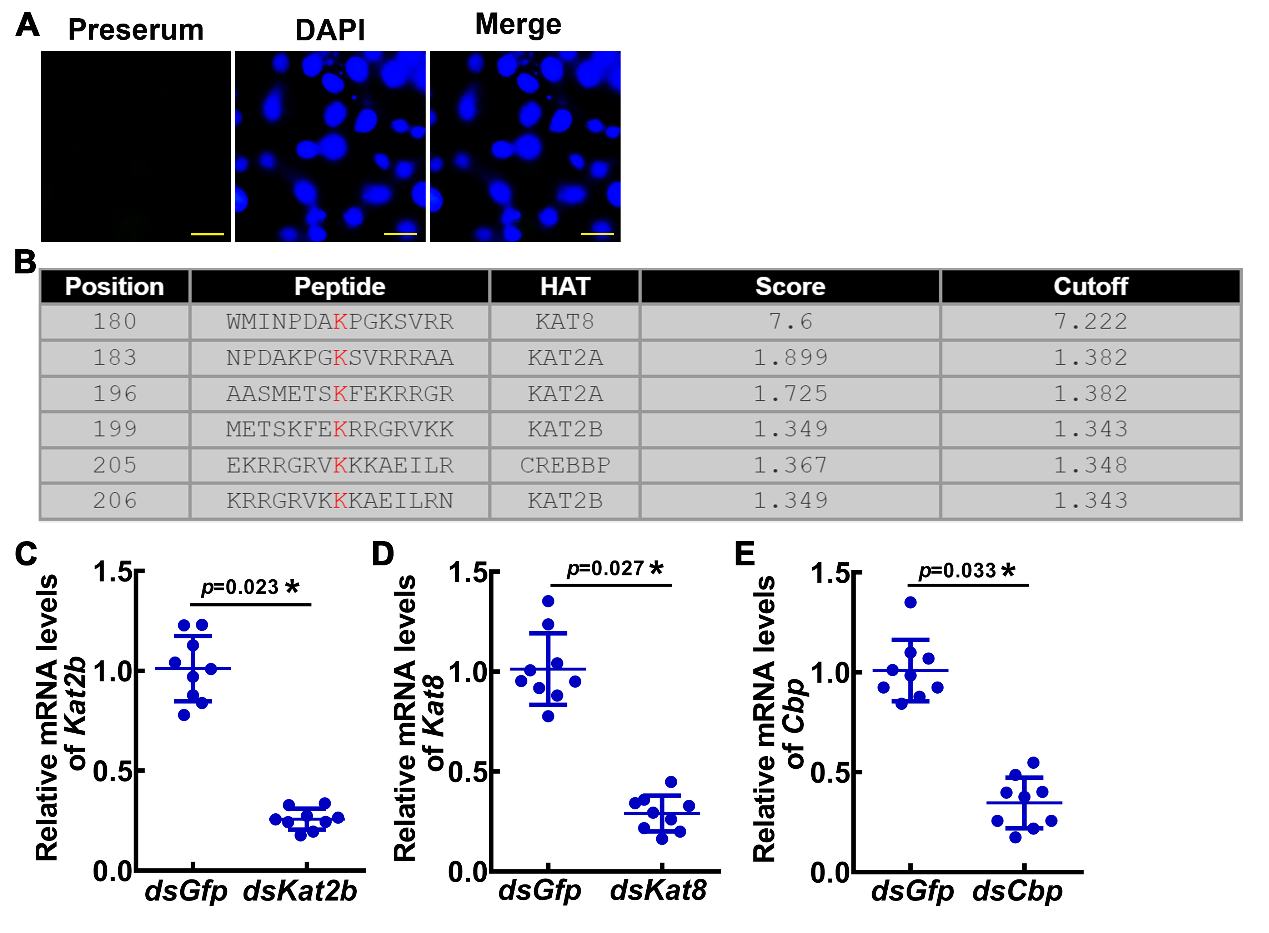


**Figure S12.** **The preserum of FOXO antibody and prediction of FOXO acetylation.** *A,* The preserum of FOXO antibody was analyzed by immunocytochemistry. Blue showing nuclei stained with DAPI. The ruler represents 20 μm. *B,* Prediction of FOXO acetylation. KAT8: *H. armigera* KAT8 XM_049841299; KAT2A/KAT2B: *H. armigera* KAT2B XM_021328550; CREBBP: *H. armigera* CBP XM_049841075. *C,* Knockdown efficiency of *Kat2b* in HaEpi cells*.* *D,* Knockdown efficiency of *Kat8* in HaEpi cells*.* *E,* Knockdown efficiency of *Cbp* in HaEpi cells*.* Data were mean ± SD of three replicates. **p* < 0.05, ***p* < 0.01 by two-tailed Student's *t* test.

**
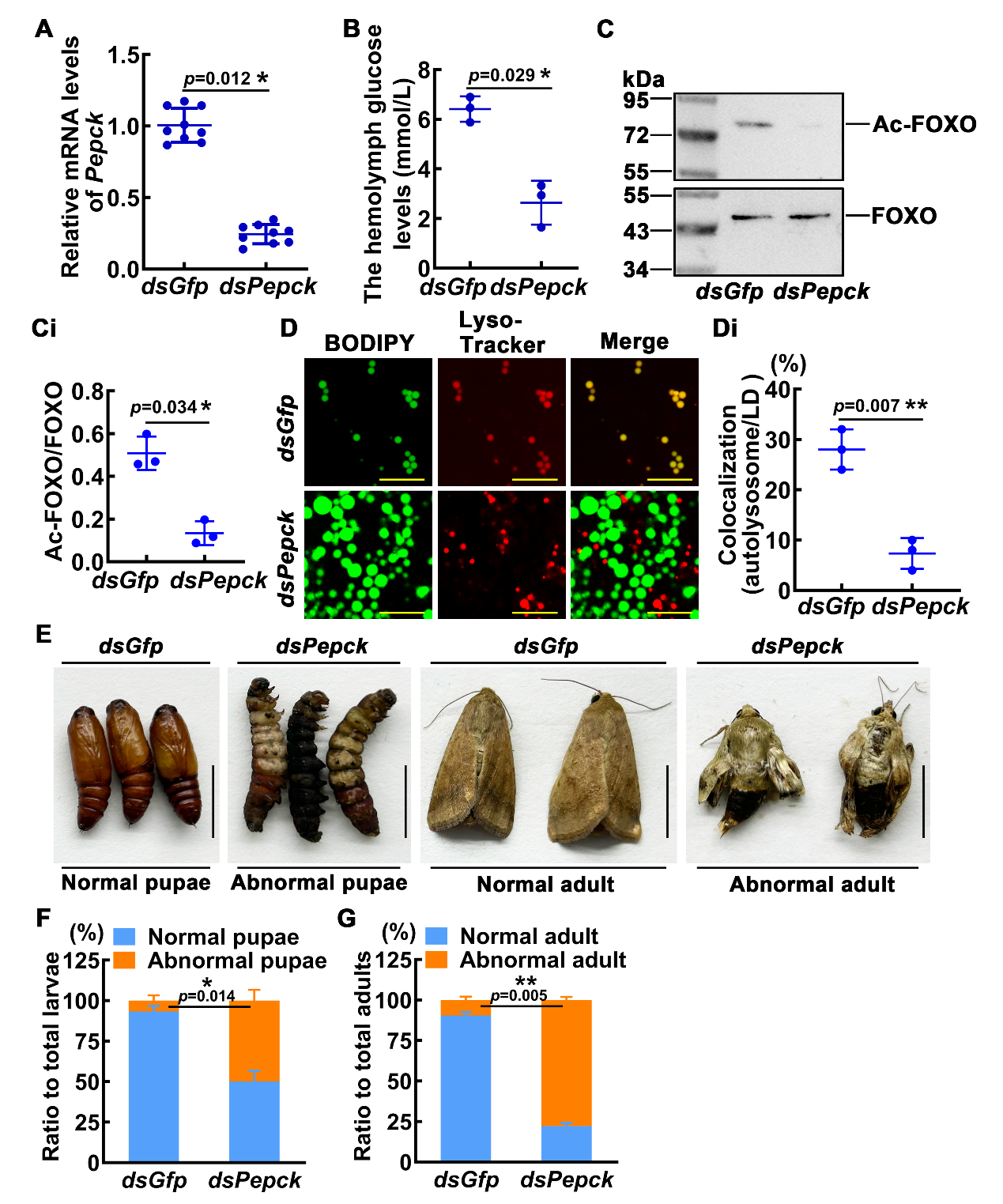
**

**Figure S13. Interference with *Pepck* inhibited FOXO acetylation and lipophagy.** *A,* Knockdown efficiency of *Pepck* in the fat body. dsRNA injection into hemocoel at larva of 6th-6 h (once every 24 h, with a total of four injections). Samples were taken 144 h after the first injection of dsRNA. *B,* The hemolymph glucose levels were measured after the injection of *dsPepck* or *dsGfp*. *C,* Western blotting showed the acetylation of FOXO after *Pepck* knockdown. *Ci,* The protein band density in (C) was quantified by ImageJ. *D,* The colocalization of lysosomes and LDs in fat body cells. Lysosomes were stained with Lyso-Tracker (red), and LDs were stained with BODIPY (green). Yellow indicates examples of Lyso-Tracker-positive structures containing LDs. The ruler represents 50 μm. Images were collected 144 h after the first injection of dsRNA. *Di,* Statistical analysis of autolysosome numbers in (D). *E,* Phenotypes after dsRNA injection. The ruler represents 1 cm. *F,* Ratio of phenotypes at pupation stage. *G,* The ratio of phenotypes of adults. All experiments were performed in triplicate, and statistical analysis was conducted using Student's *t* test (**p* < 0.05; ***p* < 0.01). The bars indicate the mean ± SD.


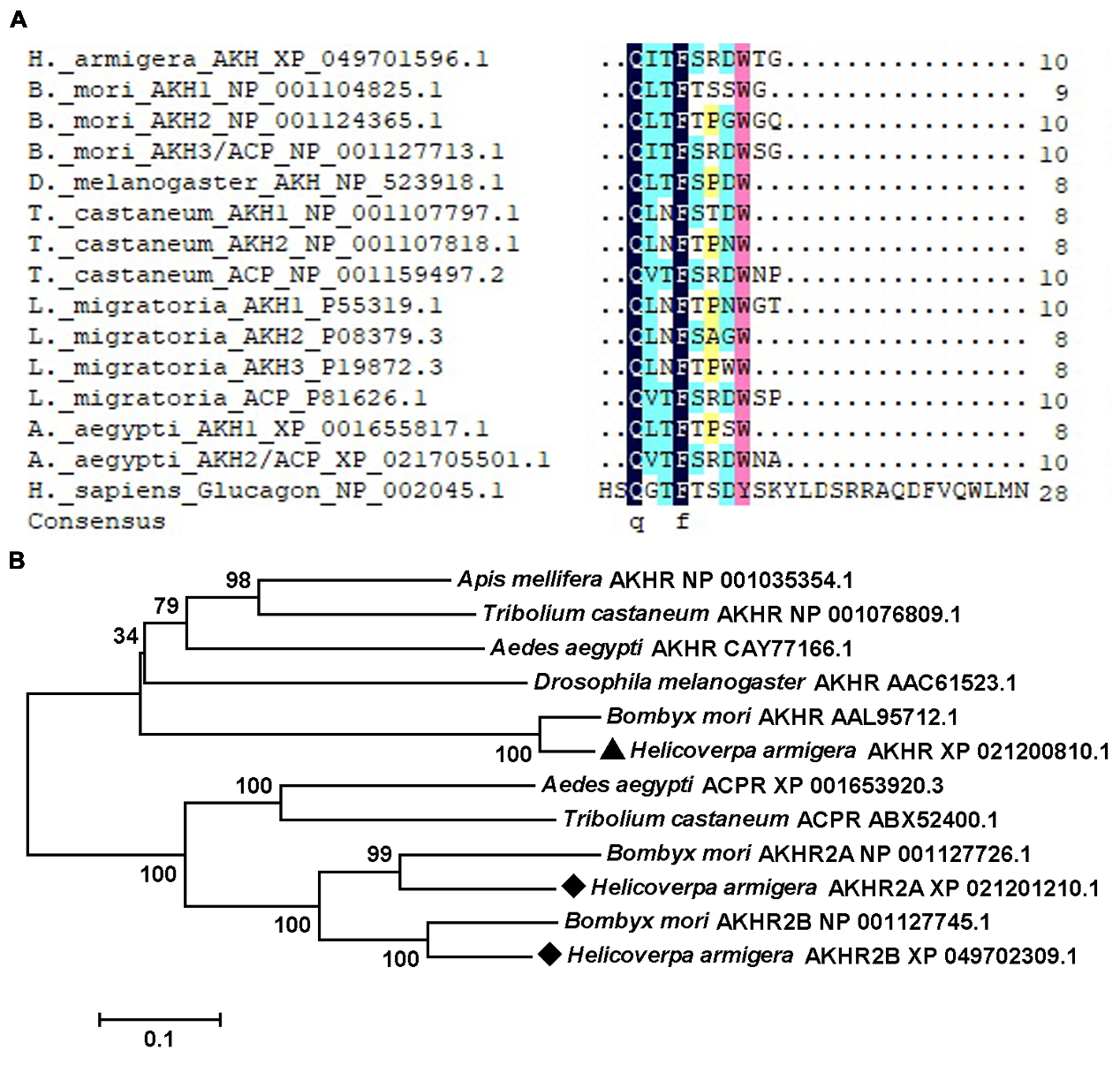


**Figure S14.** **Sequence analysis of AKH and phylogenetic tree analysis of AKHR.** *A,* The active peptide sequence analysis of AKH from different species. *B,* Phylogenetic tree analysis of AKHR from different species.


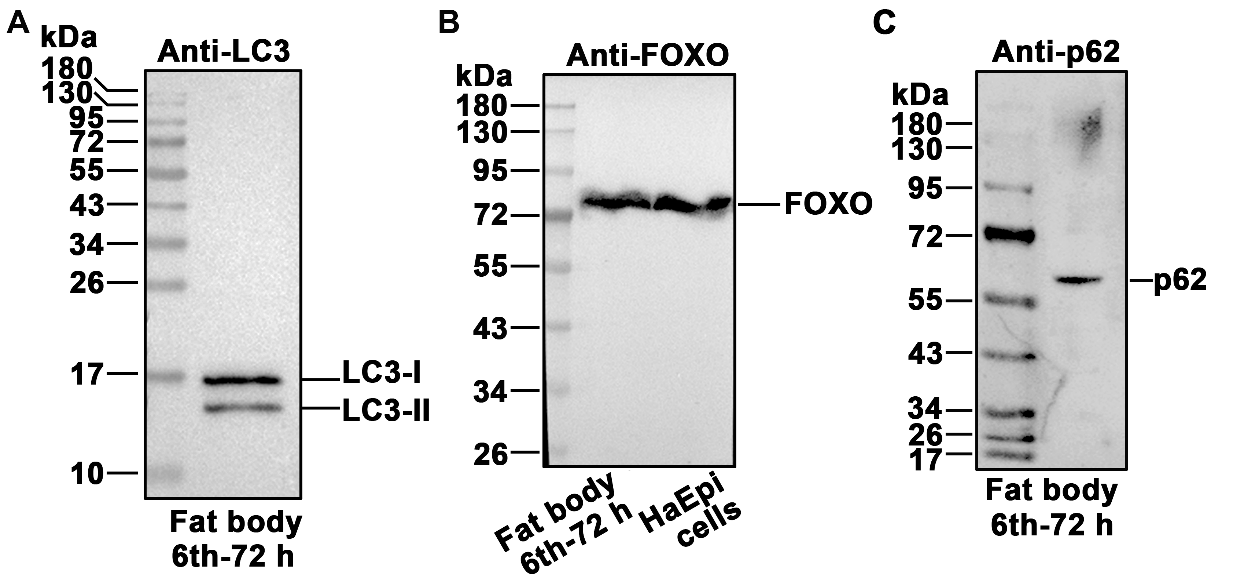


**Figure S15. Western blotting analyzed antibody specificity.** *A,* The specificity of the LC3 antibody in the fat body was analyzed by western blotting. *B,* The specificity of FOXO antibody in the fat body tissue and HaEpi cells was analyzed by western blotting. *C,* The specificity of p62 antibody in the fat body tissue was analyzed by western blotting.

**Table S1. Oligonucleotide sequences of PCR primers**

| **Primer name** | **5´ 3´ nucleotide sequence** |  |
| --- | --- | --- |
| **qRT-PCR** |  |  |
| G6pase-RTF | gatggaacaaatctacgca | XM_021331109.1 |
| G6pase-RTR | agccaccatttcataagagcatt |  |
| Pepck-RTF | cgcaagaacgatgagggcaaat | XM_021342225.1 |
| Pepck-RTR | gttgtcacagcggcgcaggat |  |
| Brummer-RTF | cgttattgtctctgagttcccc | XM_049841013.1 |
| Brummer-RTR | tagttctatgcttgtgtttgcc |  |
| Lipase3-RTF | caggataccgagcagag | XM_021337150.2 |
| Lipase3-RTR | aaggaaaagtcccaaaa |  |
| Atg1-RTF | cggaccacctccatcaa | XM_021329719.1 |
| Atg1-RTR | cactcgctccctggctttc |  |
| Atg4-RTF | atggatgccgtgtttga | XM_021327178.1 |
| Atg4-RTR | gatgtcccttctgatgc |  |
| Atg5-RTF | atggctaacgatagagaag | KT895433.1 |
| Atg5-RTR | agttagatgccatggcag |  |
| Atg7-RTF | aaatggaggtttcaaagagga | XM_021331200.1 |
| Atg7-RTR | caagtagagttgggcagtggt |  |
| Atg8-RTF | aagagaaagaccgaaggcg | XM_021325898.1 |
| Atg8-RTR | ggtctccgagtctagccttc |  |
| Atg14-RTF | atggagatagaagagttgagc | XM_021335677.1 |
| Atg14-RTR | cacatggaggcgaatgac |  |
| Casp3-RTF | acccatcaaatacgagcaatcc | XP_021187204.1 |
| Casp3-RTR | catcattgtccgtgccattcctt |  |
| Akh-RTF | ctcgactgcggacagtttacc | XM_021342224.1 |
| Akh-RTR | agctcctggtggtgcttgg |  |
| Akhr-RTF | gacgaactgcctctggacat | XM_021345135.2 |
| Akhr-RTR | agcaccgtcaagttccctgt |  |
| Kat2b-RTF | acgcaggcagaaggagattg | XM_049841299 |
| Kat2b-RTR | cggtgacagatcgacggttg |  |
| Kat8-RTF | acggcaaggaacacaagatt | XM_021328550 |
| Kat8-RTR | atagccaacgagatgtgctc |  |
| Cbp-RTF | agttgatgggaggtgcttgg | XM_049841075 |
| Cbp-RTR | ttgagctgttgctgttggga |  |
| Foxo-RTF | tcattacccaagccagcac | XM_021330987.1 |
| Foxo-RTR | tccatccagccgaagagt |  |
| Ecr-RTF | aattgcccgtcagtacga | ACD74807.1 |
| Ecr-RTR  p62-RTF  p62-RTR | tgagcttctcattgagga  cgtcattgggttccgcta  ctctcccctccacgctcg | XM_021344638.3 |
| **RNAi** |  |  |
| Brummer-RNAiF | gcgtaatacgactcactataggaatgaaagaggaatggaagg |  |
| Brummer-RNAiR | gcgtaatacgactcactataggttgtttttgatgaaagcgta |  |
| Lipase3-RNAiF | gcgtaatacgactcactatagggaccccaacagcggcaagt |  |
| Lipase3-RNAiR | gcgtaatacgactcactataggtctccgaagccatcacgaa |  |
| Akhr-RNAiF | gcgtaatacgactcactataggtcataccgttgctgtccact |  |
| Akhr-RNAiR | gcgtaatacgactcactatagggaagtcttgaaccacgcctc |  |
| Foxo-RNAiF | gcgtaatacgactcactataggcaagacaacagactcacg |  |
| Foxo-RNAiR | gcgtaatacgactcactataggttgtccgaagtccgtttg |  |
| Ecr-RNAiF | gcgtaatacgactcactatagggacgctggtataacaacggagga |  |
| Ecr-RNAiR | gcgtaatacgactcactatagggaagctggagacaactcctcacg |  |
| Kat2b-RNAiF | gcgtaatacgactcactatagggatagtgttctgcgcggtgac |  |
| Kat2b-RNAiR | gcgtaatacgactcactatagggtgcgtggttctttaccgca |  |
| Kat8-RNAiF | gcgtaatacgactcactatagggacctaatccggagtgtcgga |  |
| Kat8-RNAiR | gcgtaatacgactcactatagggggtccatctccgcgtaagac |  |
| Cbp-RNAiF | gcgtaatacgactcactatagggcagggcttctgctgtgataattg |  |
| Cbp-RNAiR  *Pepck*-RNAiF  *Pepck*-RNAiR  *Atg1*-RNAiF  *Atg1*-RNAiR  *p62*-RNAiF  *p62*-RNAiR | gcgtaatacgactcactatagggggtggatggcagtggaagata  gcgtaatacgactcactataggatgggttctttggtgttgctc  gcgtaatacgactcactataggatttgccctcatcgttcttgc  gcgtaatacgactcactatagggtctgtggtcaagggaaaa  gcgtaatacgactcactataggtgtgggtgagcaggatatt  gcgtaatacgactcactatagggtgcgatgactacgacttgtg  gcgtaatacgactcactataggtgagtttcagtttggttgggt |  |
| **Luciferase** |  |  |
| pAkh-LUC-F | ccatgattacgaattcccgggttatttactagctccaagggggat |  |
| pAkh-LUC-R | tttggcgtcttccatgagctccaccatggtaactgcgaacttaaccttcttatacg |  |
| pAkhr-LUC-F | ccatgattacgaattcccgggctcacggcatactccaca |  |
| pAkhr-LUC-R | tttggcgtcttccatgagctccaccatggtcaccaaccagcacttcac |  |
| **ChIP** |  |  |
| Akh-ECRE1-F | tgataaataatgaatctgaggtc |  |
| Akh-ECRE1-R | gtaaaaactgttaaataagccaa |  |
| Akh-ECRE2-F | taggctaacccggtagtttatca |  |
| Akh-ECRE2-R | cgtctttgtctggtcgtctgtat |  |
| Akh-FOXOBE1-F | aggggaataactgaattaacatc |  |
| Akh-FOXOBE1-R | aaatagaacataatgccaacaga |  |
| Akh-FOXOBE2-F | attaaataatagttagggaggg |  |
| Akh-FOXOBE2-R | tagtaagccagtgagtcagaga |  |
| Akhr-ECRE1-F | tatgtaattgcattattgcagg |  |
| Akhr-ECRE1-R | gaaagtcaaaagaacgtctcga |  |
| Akhr-ECRE2-F | gattcgagacgttcttttgact |  |
| Akhr-ECRE2-R | aaatagctttggctttttttac |  |
| Akhr-FOXOBE1-F | tgactaacatctatcgaaagatc |  |
| Akhr-FOXOBE1-R | attaaaacaaagtttaaccaaat |  |
| Akhr-FOXOBE2-F | ttgtcaacaaaatccaaggga |  |
| Akhr-FOXOBE2-R | aatgatgagagctgcaaaggt |  |

1. *Corresponding author: Xiao-Fan Zhao, E-mail address: [xfzhao@sdu.edu.cn](mailto:xfzhao@sdu.edu.cn) [↑](#footnote-ref-1)
